# Temporal analysis reveals a key role for *VTE*5 in vitamin E biosynthesis in olive fruit during on-tree development

**DOI:** 10.3389/fpls.2015.00871

**Published:** 2015-10-21

**Authors:** Egli C. Georgiadou, Thessaloniki Ntourou, Vlasios Goulas, George A. Manganaris, Panagiotis Kalaitzis, Vasileios Fotopoulos

**Affiliations:** ^1^Department of Agricultural Sciences, Biotechnology and Food Science, Cyprus University of TechnologyLemesos, Cyprus; ^2^Department of Horticultural Genetics and Biotechnology, Mediterranean Agronomic Institute of ChaniaChania, Greece

**Keywords:** *Olea europaea*, developmental stages, gene expression, tocopherols, tocotrienols, tocochromanols, phytol kinase

## Abstract

The aim of this work was to generate a high resolution temporal mapping of the biosynthetic pathway of vitamin E in olive fruit (*Olea europaea* cv. “Koroneiki”) during 17 successive on-tree developmental stages. Fruit material was collected from the middle of June until the end of January, corresponding to 6–38 weeks after flowering (WAF). Results revealed a variable gene regulation pattern among 6–38 WAF studied and more pronounced levels of differential regulation of gene expression for the first and intermediate genes in the biosynthetic pathway (*VTE5, geranylgeranyl reductase, HPPD, VTE2, HGGT* and *VTE3*) compared with the downstream components of the pathway (*VTE1* and *VTE4*). Notably, expression of *HGGT* and *VTE2* genes were significantly suppressed throughout the developmental stages examined. Metabolite analysis indicated that the first and intermediate stages of development (6–22 WAF) have higher concentrations of tocochromanols compared with the last on-tree stages (starting from 24 WAF onwards). The concentration of α-tocopherol (16.15 ± 0.60−32.45 ± 0.54 mg/100 g F.W.) were substantially greater (up to 100-fold) than those of β-, γ-, and δ-tocopherols (0.13 ± 0.01−0.25 ± 0.03 mg/100 g F.W., 0.13 ± 0.01−0.33 ± 0.04 mg/100 g F.W., 0.14 ± 0.01−0.28 ± 0.01 mg/100 g F.W., respectively). In regard with tocotrienol content, only γ-tocotrienol was detected. Overall, olive fruits (cv. “Koroneiki”) exhibited higher concentrations of vitamin E until 22 WAF as compared with later WAF, concomitant with the expression profile of phytol kinase (*VTE5*), which could be used as a marker gene due to its importance in the biosynthesis of vitamin E. To the best of our knowledge, this is the first study that explores the complete biosynthetic pathway of vitamin E in a fruit tree crop of great horticultural importance such as olive, linking molecular gene expression analysis with tocochromanol content.

## Introduction

Olive tree products are essential elements of Mediterranean diet (Ziogas et al., 2010; Anastasopoulos et al., 2011). Olive fruit is highly enriched in antioxidants such as vitamin E, carotenoids, and phenolic compounds (Aliakbarian et al., 2009; Muzzalupo et al., 2011; Goulas et al., 2012), which are known to provide several health-promoting benefits and reduce the risk of chronic diseases (Aliakbarian et al., 2009). Despite its relatively small fruit size, “Koroneiki” represents *ca*. 60% of the total olive-growing area in Greece due to its high yield of high quality olive oil (Anastasopoulos et al., 2011), thus rendering it a model cultivar for further studies.

The olive fruit on-tree developmental phases can be distinguished into five interrelated stages. The first one refers to flowering, fertilization and fruit set. During this phase rapid, early cell division takes place, which enhances embryonic development. The second stage concerns the growth of the seed, which includes intense cell division, resulting in the development of the endocarp (seed/pit) and in the slight growth of the mesocarp (flesh). The hardening of the seed/pit occurs during the third stage, while during the fourth stage, the mesocarp develops and the pre-existing flesh cells expand and oil is accumulated. Ripening is the fifth stage when the fruit changes color from dark green to lighter green/purple and the softening process is initiated (Conde et al., 2008; Alagna et al., 2009).

Tocochromanols are comprised of eight forms which are divided in two groups with four forms each, namely α-, β-, γ-, and δ-tocopherols and tocotrienols, respectively.

Tocopherols and tocotrienols are well-known as lipophilic bioactive compounds. Tocotrienols have been reported to exert a protective effect against cancer, diabetes, and cardiovascular and neurological diseases (Aggarwal et al., 2010). In addition, tocochromanols are considered powerful natural antioxidants; however, tocotrienols are more potent antioxidants than tocopherols due to the presence of conjugated double bonds in the hydrophobic side chain (Colombo, 2010). Further evidence also supports that tocotrienols offer health benefits as they are antioxidant, maintain the cardiovascular system and protect against cancer and other illness (Nesaretnam et al., 2007; Nesaretnam, 2008). Existing literature shows that α-tocopherol is the most abundant in olive fruit reaching maximum concentration of ~89% of total tocopherol content depending on the cultivar and developmental stage, with other tocopherols in olive fruit cultivars reaching maximum concentration ~14% for β-tocopherol, ~42% for γ-tocopherol, and ~26% for δ-tocopherol (Hassapidou and Manoukas, 1993; Bruno et al., 2009; Muzzalupo et al., 2011; Bodoira et al., 2015). Tocochromanols are synthesized only by photosynthetic organisms and are potent antioxidants with a direct scavenging effect on cellular reactive oxygen species (ROS; Conde et al., 2008; Dellapenna and Mene-Saffrane, 2011).

**Figure 1:**
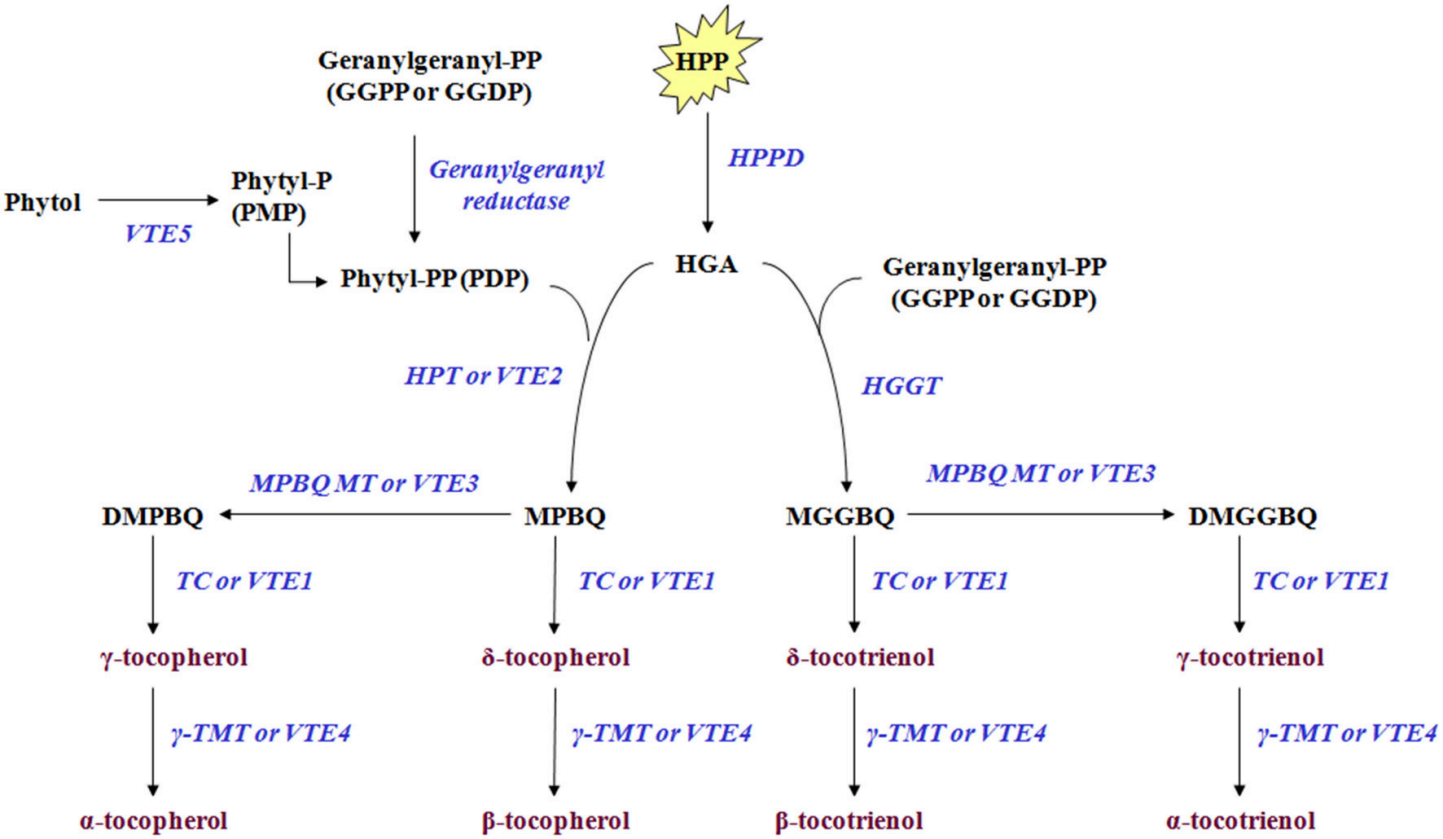
**Tocochromanol biosynthetic pathway in olive fruit**. The enzymes/genes are: HPPD, p- or 4-hydroxyphenylpyruvate dioxygenase; HPT or VTE2, homogentisate phytyltransferase or vitamin E2; geranylgeranyl reductase; VTE5, Phytol kinase or vitamin E5; Phytyl phosphate kinase; HGGT, Homogentisate geranylgeranyl transferase; MPBQ MT or VTE3, 2-methyl-6-phytyl-1,4-benzoquinol methyl transferase or vitamin E3; TC or VTE1, Tocopherol cyclase or vitamin E1; γ-TMT or VTE4, γ-tocopherol methyl transferase or vitamin E4. The metabolites are: Phytol; phytyl-P or PMP, Phytyl phosphate; phytyl-PP or PDP, Phytyl diphosphate; GGPP or GGDP, geranylgeranyl pyrophosphate = geranylgeranyldiphosphate; HPP, p- or 4-hydroxyphenylpyruvic acid; HGA, Homogentisic acid; MPBQ, 2-methyl-6-phytylbenzoquinol; DMPBQ, 2,3-dimethyl-6-phytyl-1,4-benzoquinol; MGGBQ, 2-methyl-6-geranylgeranylbenzoquinol; DMGGBQ, 2,3-dimethyl-6-geranylgeranyl-1,4-benzoquinol; α-, β-, γ-, δ-tocopherols; α-, β-, γ-, δ-tocotrienols (Figure is modified from Dellapenna and Mene-Saffrane, 2011; Ren et al., 2011; Yang et al., 2011).

Tocochromanols originate from 4-hydroxyphenylpyruvic acid (HPP) and homogentisic acid (HGA), geranylgeranyl diphosphate, phytol, and phytyl phosphate (Phytyl-P) which are derived either from the shikimate acid pathway, methylerythritol phosphate pathway (MEP) and chlorophyll degradation, respectively. The Phytyl diphosphate (Phytyl-PP) is derived either by geranylgeranyl diphosphate (GGPP) after reduction by geranylgeranyl reductase or by phytol after phosphorylation by *phytol kinase* (*VTE5*). HPP is reduced to HGA by *4-hydroxyphenylpyruvate dioxygenase* (*HPPD*) which serves as a shared precursor for the biosynthesis of tocopherols and tocotrienols (Figure 1, modified from Dellapenna and Mene-Saffrane, 2011; Ren et al., 2011; Yang et al., 2011). HGA is further decarboxylated and then condensated with a phytyl diphosphate (Phytyl-PP) into 2-methyl-6-phytylbenzoquinol (MPBQ) by *homogentisate phytyl transferase* (*VTE2*). The MPBQ is either catalyzed into 2,3-dimethyl-6-phytyl-1,4-benzoquinol (DMPBQ) by 2*-methyl-6-phytyl-1,4-benzoquinol methyl transferase* (*VTE3*) and then to γ-tocopherol by *tocopherol cyclase* (*TC* or *VTE1*) or to δ-tocopherol directly by *TC*. The γ- and δ- tocopherols are transformed into α- and β- tocopherols by γ*-tocopherol methyl transferase* (γ*-TMT* or *VTE4*). Alternatively, the HGA is metabolized into *2-methyl-6-geranylgeranylbenzoquinol* (*MGGBQ*) by *homogentisate geranylgeranyl tranferase* (*HGGT*) and then to δ- and subsequently β- tocotrienol catalyzed by *tocopherol cyclase* (*VTE1*) and γ*-tocopherol methyl transferase* (*VTE4*), respectively. The biosynthesis of γ- and subsequently α-tocotrienol requires an additional step; the conversion of MGGBQ into 2,3-dimethyl-6-geranylgeranyl-1,4-benzoquinol (DMGGBQ) in addition to the reactions catalyzed by *VTE1* and *VTE4* (Dellapenna and Mene-Saffrane, 2011; Ren et al., 2011; Yang et al., 2011). The biosynthetic pathway of vitamin E has previously been briefly investigated on other plants such as *Solanum lycopersicum* (Quadrana et al., 2013), *Arabidopsis thaliana* (Li et al., 2010; Zhang et al., 2013, 2014), *Nicotiana tabacum* (Yabuta et al., 2013), and *Lactuca sativa* (Ren et al., 2011; Yabuta et al., 2013).

In this study, the high resolution temporal expression profiles of tocopherol and tocotrienol biosynthetic genes were determined in parallel with the content of the four tocopherol and four tocotrienol forms during on-tree olive fruit developmental stages. These developmental programs in olive fruit are comprised of a period of 8 months starting in June and ending in January. Results suggest a high level of transcriptional regulation of α-tocopherol biosynthesis in mesocarp fruit tissue; furthermore, the regulatory importance of the first steps in biosynthetic pathway is highlighted.

## Material and methods

### Fruit material and experimental design

This study was conducted using olive fruits (cv. “Koroneiki”) during 17 different developmental stages, designated as *S*_1_–*S*_17_. These stages correspond to 6–38 weeks after flowering (WAF). Supplementary Table 1 provides a detailed list of the harvesting days and weeks after flowering. Fruit material was harvested from the Experimental Farm at the Mediterranean Agronomic Institute of Chania, Crete. Detailed meteorological data (air temperature, rainfall, and air relative humidity) in the experimental orchard during the 38 weeks after flowering are shown in Supplementary Figure 1. Olive fruits were collected at 1.7 m height around the tree canopy (symmetrically around the crown) of four trees with similar bearing habits and maturation. Approximately 25 olive fruits per tree were collected, pooled and transferred into the laboratory. Olive fruits were grouped based on the phenological growth stages in accordance with the BBCH (Biologische Bundesanstalt, Bundessortenamt, Chemische Industrie) scale (Sanz-Cortes et al., 2002; see Supplementary Table 2). The mesocarp developmental stage corresponds to 6–22 WAF, while the ripening stage of the olive fruit corresponds to 22–38 WAF (Conde et al., 2008; Alagna et al., 2009). Subsequently, fruit weight and diameter (*n* = 100) was monitored (Supplementary Figure 2). Dry content was also determined: *ca*. 3.5 g of olives fruits were dried in a forced air oven at 65°C for about 3–4 days to constant weight, based on which % humidity was calculated (Supplementary Figure 3). The rest of the olives were washed with household bleach:ddH_2_O (1:1) for 3 min and rinsed several times (5–6) with ddH_2_O. Subsequently, the olive fruit were separated from its olive seed. The flesh was cut and put immediately in liquid nitrogen, ground and kept at −80°C until needed as elsewhere described (Beltran et al., 2004).

### RNA extraction and rDNase treatment

Total RNA was extracted from three independent tissue samples of 100 mg olive fruit material per fruit developmental stage following the protocol described by Christou et al. (2014). RNA was treated with RNase-free DNase (Cat. No. 04716728001, Roche), in order to completely remove gDNA. Subsequently, ddH_2_O was added to a final volume of 300 μL, along with an equal volume of chloroform, mixed in a vortex, and centrifuged on bench-top centrifuge (Eppendorf Centrifuge 5415 R) at 16000 × g for 30 min at 4°C. The upper phase was transferred to a new, cold tube. Finally, 2.5 volumes of absolute ethanol and 1/10 volume 3 M CH_3_COONa (pH 4.8) was added for precipitation. The sample was mixed, incubated at −80°C overnight and centrifuged at 16000 × g for 30 min at 4°C. The supernatant was discarded and incubated at 50°C for 2–3 min. RNA was dissolved in 20 μL ddH_2_O. The RNA integrity was analyzed spectrophotometrically (Nanodrop 1000 Spectrophotometer, Thermo Scientific), confirmed with gel electrophoresis and stored at −20°C until use.

### cDNA synthesis and real-time RT-PCR analysis

For first-strand cDNA synthesis, 1 μg of total RNA from each RNA extraction was converted into cDNA using the Primescript 1st Strand cDNA synthesis kit according to the manufacturer's instructions (Takara Bio, Japan). Real-time PCR was also performed with Biorad IQ5 real-time PCR cycler (Biorad, USA). In total, three biological replicates were performed for each developmental stage. The reaction mix contained 4 μL cDNA in reaction buffer (5-fold diluted first-strand cDNA), 0.5 μL of each primer (10 pmol/μl; Supplementary Table 4) and 5 μL 2X master mix (KAPA SYBR® FAST qPCR Kit, Kapa-Biosystems). The total reaction volume was 10 μL. The initial denaturation stage was at 95°C for 5 min, followed by 40 cycles of amplification [95°C for 30 s, annealing temperature (Tm °C) for 30 s, and 72°C for 30 s] and a final elongation stage at 72°C for 5 min. Gene amplification cycle was followed by a melting curve run, carrying out 61 cycles with 0.5°C increment between 65 and 95°C. The annealing temperature of the primers used ranged between 54 and 65°C as shown in Supplementary Table 4. The *UBQ2* gene was used as a housekeeping reference gene.

Previously published oligonucleotide primers were used for olive *geranylgeranyl reductase* (Muzzalupo et al., 2011) and *UBQ2* (Hernández et al., 2009). A search for Expressed Sequence Tag (EST) homologs of the other seven genes (*VTE5, HPPD, VTE2, HGGT, VTE3, VTE1*, and *VTE4*) was conducted based on the NCBI database (http://www.ncbi.nlm.nih.gov/). The ESTs with the highest score were subsequently queried at the eudicotyledons databases of the TIGR Plant Transcript Assemblies (http://plantta.jcvi.org/index.shtml). The ESTs with the highest similarity were chosen for further search at the OLEA EST db (http://140.164.45.140/oleaestdb/search.php) that includes 454 pyrosequencing data (Alagna et al., 2009; Supplementary Table 3). For the selected ESTs from OLEA EST db, oligonucleotide primer sets used were designed using Primer 3 (http://frodo.wi.mit.edu/; Supplementary Table 4). The length of all PCR products ranged from 100–300 bp. *Ct*-values, means, and standard deviations for all samples/time-points examined are presented in Supplementary Table 12.

### Phylogenetic analysis

The *Olea europaea VTE5, HPPD, VTE2, HGGT, VTE3, VTE1*, and *VTE4* amino acid residues were queried for homology against known proteins in the NCBI database, employing the Blastp algorithm (http://blast.ncbi.nlm.nih.gov/Blast.cgi). Around thirty proteins that had similarity to the olive *VTE5, HPPD, VTE2, HGGT, VTE3, VTE1*, and *VTE4* (and were also characterized as *VTE5, HPPD, VTE2, HGGT, VTE3, VTE1*, and *VTE4*) were selected in order to construct a phylogenetic tree. An amino acid alignment was conducted using the MUSCLE algorithm and all positions that had gaps were removed using alignment curation. The Maximum Likelihood (ML) method and an approximate Likelihood-Ratio Test (aLRT) were selected for the construction of the dendrogram and for statistical support testing of branch lengths. All the above procedures were conducted using the “A la Carte” workflow as implemented in the http://phylogeny.lirmm.fr/phylo_cgi/index.cgi site, as reported by Dereeper et al. (2008). Visualization of the tree was possible via the Treeview software (Page, 1996).

### Chromatographic determination of tocochromanols

For the recovery of tocopherols and tocotrienols, approximately 100 mg of olive fruit was extracted with 1 ml acetonitrile-methanol-water (72/18/10, v/v/v) in 2-ml Eppendorf tube. The mixture was shaken for 15 min at 60°C in Lab Companion SI-600R benchtop shaker in the dark following 5 min pre-incubation. Then, the mixture was centrifuged on bench-top centrifuge at 16000 × g for 5 min at 4°C (Eppendorf Centrifuge 5415 R) and the supernatant was collected and stored at −20°C until HPLC analysis (Gruszka and Kruk, 2007). Three biological replicates were performed for each developmental stage.

A Waters series HPLC (Model “e2695”) equipped with vacuum degasser, quaternary pump, autosampler, thermostatted column compartment, multi λ fluorescence detector, and Empower software (Waters Corporation, Milford, Ireland) for data collection and analysis was used. After filtration on Millipore paper (0.22 μm), 20 μL of each extract were injected on a reverse phase XTerra RP18 (5 μm; 4.6 × 250 mm) column (Waters Corporation, Milford, Ireland). An isocratic elution was also performed using a mobile phase composed of acetonitrile/ methanol/ 2-propanol (40/55/5, v/v/v) at a flow rate of 0.8 ml min^−1^. The fluorescence detector was set at an excitation wavelength of 292 nm and an emission wavelength of 335 nm (Tsochatzis et al., 2012).

A six-level calibration curve was constructed for each of the studied tocochromanols, with triplicate determinations at each level. The chromatographic peaks were identified by the retention times of the standard compounds.

### Statistical analysis

All real-time RT-PCR data analyses were performed using the REST-XL software according to Pfaffl et al. (2002) for relative quantification of gene expression and statistical analysis (pairwise fixed reallocation randomization test). The 6 week after flowering (WAF) sampling point was used for calibrating gene expression values.

Statistical analysis of the results from the HPLC-Fluorescence detector analysis was carried out using the software package SPSS v17.0 (SPSS Inc., Chicago, USA) and the comparison of averages of each treatment was based on the analysis of variance (One-way ANOVA) according to Duncan's multiple range test at significance level 5% (*P* ≤ 0.05).

## Results

### *In silico* analysis of genes involved in the biosynthetic pathway of vitamin E

BLAST analysis on NCBI and OLEA EST db databases resulted in the identification of single cDNAs for each of *VTE5, HPPD, geranylgeranyl reductase, VTE2, HGGT, VTE3, VTE1*, and *VTE4*. The deduced amino acid sequences of *VTE5, HPPD, VTE2, HGGT, VTE3, VTE1*, and *VTE4* reveal high similarities with homologs of other plant species, suggesting that the components of the pathway are highly conserved between plants (Supplementary Tables 5–11). This was further illustrated following phylogenetic analysis of all examined components of the pathway, where olive tocochromanol biosynthesis genes grouped together with other genes of similar function from other dicotyledonous plants (Supplementary Figures 4–10).

### Quantification of gene expression profiles during fruit development and ripening

**Figure 2:**
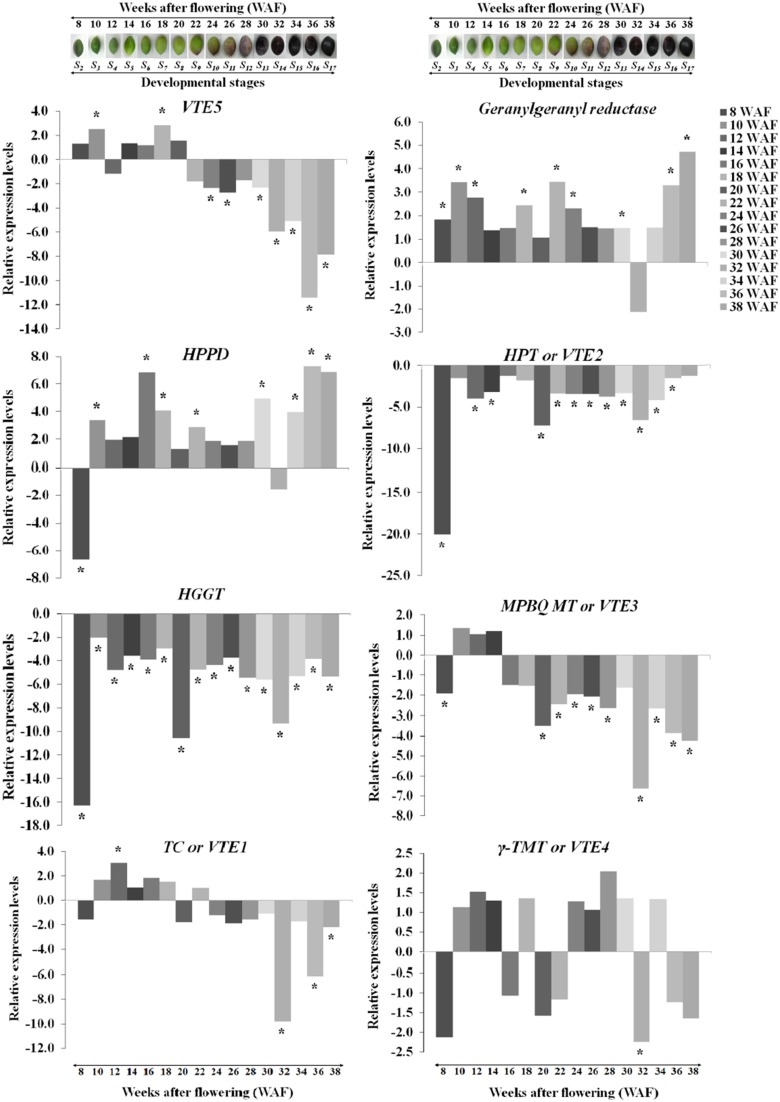
**Relative expression levels of vitamin E biosynthesis genes (***VTE5, geranylgeranyl reductase, HPPD, VTE2, HGGT, VTE3, VTE1***, and ***VTE4***) in olive fruit (cv. “Koroneiki”) during 6–38 WAF (***n*** = 3)**. The olives on top demonstrate the phenotypes of olive fruit at the different sampling stages. Values that differ from the control (6 WAF) with significance level *P* ≤ 0.05 are marked with ^*^. Data are based on a statistical analysis of the means of three replications (Pfaffl et al., 2002).

Transcript abundance of *VTE5, geranylgeranyl reductase, HPPD, VTE2, HGGT, VTE3, VTE1*, and *VTE4* was determined using a qRT-PCR approach. The expression of *VTE5* was up-regulated during 8–20 WAF of fruit development with higher levels observed at 10 WAF and 18 WAF (Figures 2, **5**). At the 22 WAF, a significant drop in expression was detected. This drop was lower than the calibrator of 6 WAF levels and sustained up to 34 WAF, while it further decreased for the over-ripe stages of olive fruit.

*Geranylgeranyl reductase* mRNA expression was higher compared with 6 WAF throughout fruit development and ripening, with highest levels at 10, 36, and 38 WAF. A decrease in expression was detected only after 32 WAF. Similar expression pattern was observed also for *HPPD* with peaks in expression after 16 and 30 WAF, while only two stages (8 and 32 WAF), exhibited a suppressed expression profile. The highest expression levels were at 16, 36, and 38 WAF. Transcript levels of *VTE2* and *HGGT* were down-regulated throughout fruit development and ripening with the highest decrease for both genes after 8, 20, and 32 WAF.

Different patterns of expression were observed for *VTE3* and *VTE1* with up-regulation observed during most of the developmental stages and down-regulation during late stages of development and the entire ripening process. Specifically, *VTE3* mRNA levels were lower compared with the calibrator (6 WAF) after 8 WAF and increased up to 14 WAF; thereafter, its expression went descending, culminating during the over-ripe stages. *VTE1* expression increased after 10 WAF all the way to 18 WAF, as well as after 22 WAF while it decreased thereafter up to the 38 WAF. This pattern of transcript abundance resembles the pattern of both *VTE3* and *VTE5*. The highest suppression levels were also observed after 32 WAF. *VTE4* expression was increased during 10–14 WAF and decreased at 8, 16, 20, and 22 WAF. Similarly, up-regulation was observed during the early stages of fruit ripening and down-regulation thereafter with the exception of 34 WAF. The pattern of *VTE4* expression can be considered unique among all the genes involved in the vitamin E pathway.

### Quantification of tocopherols and tocotrienols during fruit development and ripening

**Figure 3:**
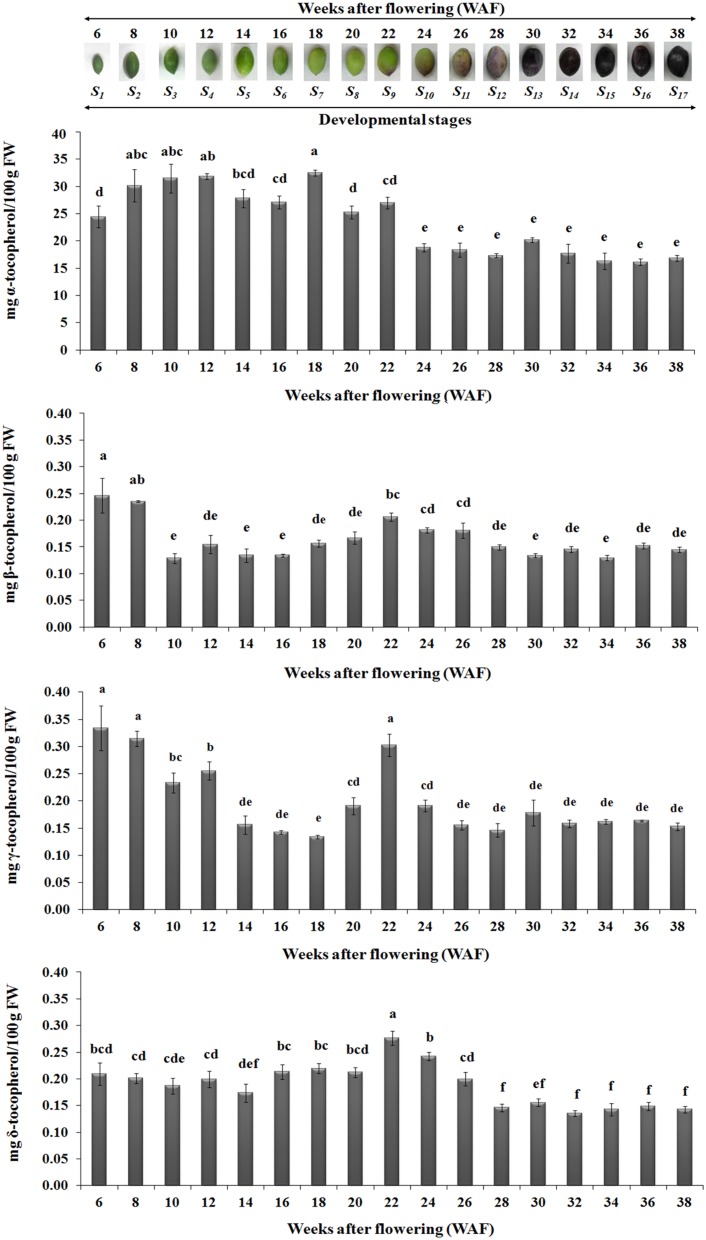
**Tocopherol content in olive fruit (cv. “Koroneiki”) during 6–38 WAF (***n*** = 3)**. The olives on top demonstrate the phenotypes of olive fruit at the different sampling stages. Values followed by the same letter are not significantly different according to Duncan's multiple range test at significance level 5% (*P* ≤ 0.05). Data are the means of three replications ± SE.

**Figure 4:**
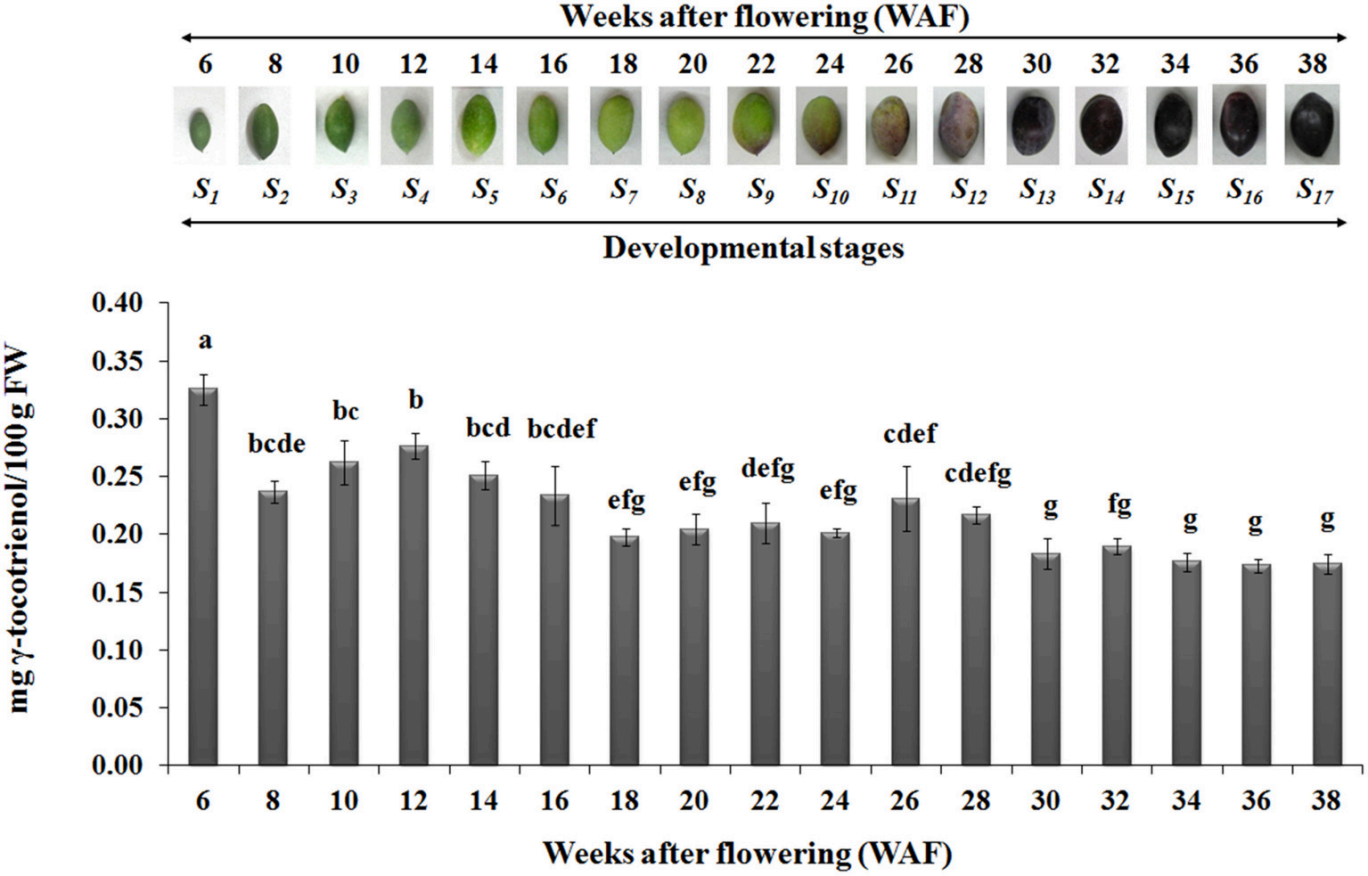
**Gamma-tocotrienol content in olive fruit (cv. “Koroneiki”) during 6–38 WAF (***n*** = 3)**. The olives on top demonstrate the phenotypes of olive fruit at the different sampling stages. Values followed by the same letter are not significantly different according to Duncan's multiple range test at significance level 5% (*P* ≤ 0.05). Data are the means of three replications ± SE.

**Figure 5:**
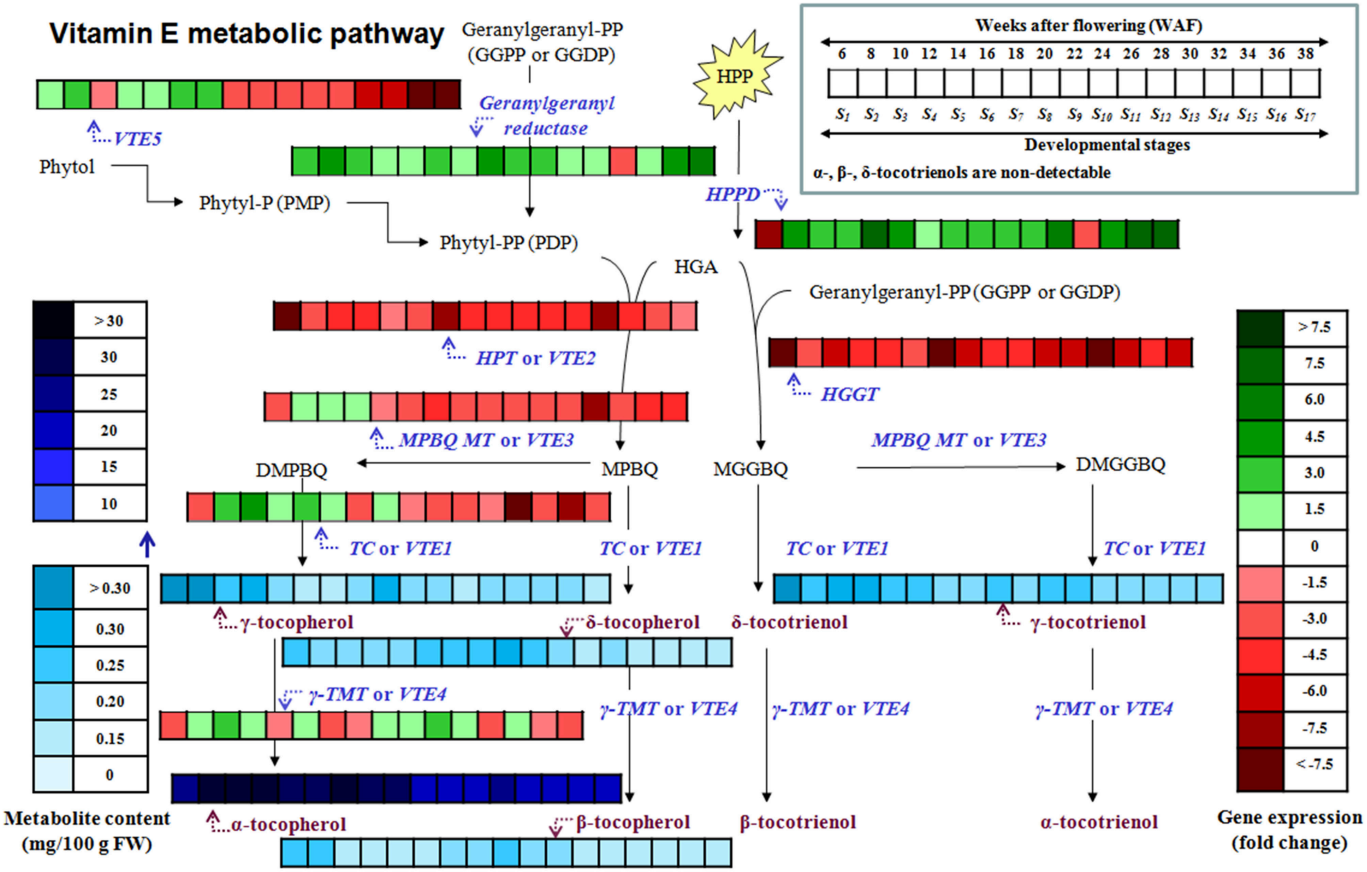
**Heat map of the metabolite content and relative expression levels of genes in the biosynthetic pathway of vitamin E of olive fruit (cv. “Koroneiki”) during 6–38 WAF**. α-, β-, δ-tocotrienols are non-detectable.

The abundance of α, β, γ, and δ forms of tocochromanols was determined on 17 successive developmental stages of fruit development and on-tree ripening in cv. “Koroneiki” in order to study their temporal variation. All forms of tocopherols and one form of tocotrienol (γ-tocotrienol) were detected (Figures 3–**5**). Alpha-tocopherol was the most abundant form of tocochromanols in accordance with previous studies (e.g., Bodoira et al., 2015). The highest concentrations of α-tocopherol were observed within the period 6–22 WAF with maximum content detected at 18 WAF (32.45 ± 0.54 mg/100 g F.W.). Alpha-tocopherol content was then stabilized at lower concentrations at the early stages of fruit ripening, after 22 WAF, and remained at those concentrations up to 38 WAF (16.86 ± 0.53 mg/100 g F.W.). Similar patterns of abundance were also observed for δ- tocopherol with a shift to lower concentrations after 26 WAF (0.20 ± 0.01 mg/100 g F.W.), while the highest concentration was detected at 22 WAF (0.28 ± 0.01 mg/100 g F.W.).

Highest concentrations of β- tocopherol were observed at the early stages of fruit development, after 6 and 8 WAF, and fruit ripening, after 22, 24, and 26 WAF. Low concentrations of β- tocopherol were quantified between 10–20 WAF and 28–38 WAF. γ-tocopherol exhibited similar fluctuations in content to those observed for β- tocopherol.

In regard with tocotrienol content, only γ-tocotrienol was detected, displaying increased concentrations between 6 and 28 WAF with the highest concentration being observed at 6 WAF (0.33 ± 0.01 mg/100 g F.W.). Finally, the concentrations of all tocopherols and γ-tocotrienol were lowered after 24 WAF when the ripening process was initiated, concomitant with the recorded increase in rainfall, relative humidity and reduction of air temperature in the experimental orchard (Supplementary Figure 1).

## Discussion

Temporal biosynthesis of tocochromanols in olive fruit was explored using a combined analytical/molecular approach during on-tree development and ripening. Molecular gene expression analysis revealed highly differential levels of regulation during 6–38 weeks after flowering (WAF). Results revealed pronounced levels of differential regulation of gene expression for the first and intermediate genes in the biosynthetic pathway (*VTE5, geranylgeranyl reductase, HPPD, VTE2, HGGT*, and *VTE3*) compared with downstream components of the pathway (*VTE1* and *VTE4*). Notably, expression of *HGGT* and *VTE2* genes is significantly suppressed in all the weeks after flowering (Figure 2).

*VTE5* was up-regulated during the period of mesocarp development until 22 WAF followed by marked down regulation at the breaker stage and throughout ripening starting from 24 WAF. Similar transition in concentration was observed for tocopherols and tocotrienols with significantly higher amounts until the breaker stage (22 WAF) and much lower thereafter indicating tight correlation with the expression profile of *VTE5*. These results are in agreement with those reported in tomato fruit (Quadrana et al., 2013), which present a decrease in *VTE5* gene expression associated with tomato ripening. This decrease, directly limits phytol diphosphate [Phytyl-PP (PDP)] input supply toward VTE biosynthesis (Quadrana et al., 2013) and correlates with the low concentrations of tocopherols and tocotrienols in the olive fruit during ripening (starting from 24 WAF). These results suggest that *VTE5* is particularly important in the biosynthesis of vitamin E in olive fruit and is thus proposed as a marker gene in relevant studies.

The highest levels of expression among all all genes examined were detected for *HPPD* during not only mesocarp development but also ripening without any similar expression trend to VTE5 at the breaker stage. At the same time, *geranylgeranyl reductase* appears to be induced as well possibly in order to counter-balance the down-regulation of *VTE5*.

A key observation in the metabolite/transcript profile was that the highest levels of up-regulation of *VTE5* were detected at 18 WAF, concomitant with highest levels of α-tocopherol content (Figures 2, 3, **5**). Interestingly, olive fruit reaches 90% of its final size at that point, thus rendering it suitable for harvesting green as this point would coincide with maximum α-tocopherol content. Furthermore, oil production in the olive fruit increases and reaches a maximum at “breaker stage” (color turned from green to purple, 22 WAF) (Conde et al., 2008; Alagna et al., 2009; Bodoira et al., 2015). Present results indicate that vitamin E content increases and reaches its maximum levels at 22 WAF (breaker stage), providing evidence that *VTE5* plays an important role in olive tocochromanol biosynthesis. Conversely, the lowest expression levels of *VTE5* was detected at 36 WAF and as a result low concentrations of all tocochromanols were observed, both likely being affected by over-ripening. Numerous reports have previously shown that olive oil synthesis starts after pit hardening, showing an increase in oil production and phenolic fraction and reaching its highest levels towards the end of the mesocarp development, concomitant with the initiation of color change (Conde et al., 2008; Alagna et al., 2009; Sakouhi et al., 2011; Bodoira et al., 2015). Furthermore, during the mesocarp development, carbohydrate metabolism (glycolysis/glyconeogenesis, citrate cycle, and fructose, manose and galactose metabolism) is more prevalent.

In addition, increasing vitamin E content and oil production occurs during mesocarp development (6–22 WAF), while highest amounts are reached at breaker stage (22 WAF). During this period there is also a decrease in chlorophyll content in fruit mesocarp (Alagna et al., 2009). In contrast, olive oil production decreases, after 22 WAF along with vitamin E content. The processes of carbohydrate metabolism, fatty acid biosynthesis, and triacylglycerols (TAGs) are more evident at the beginning of the color change phase (22 WAF) and may have an effect on oil and vitamin E content. Phytol can be transformed in Phytyl-P, then in Phytyl-PP, finally producing chlorophyll, phylloquinone (vitamin K) and tocopherols. In addition, the active fatty acyl group can restrict the free phytol through the reaction of acyltransferase and produce fatty acid phyrol ester synthesis (Ischebeck et al., 2006). It has been reported that the majority of phytyl-PP for tocopherol biosynthesis in Arabidopsis seeds is derived from chlorophyll degradation (Ischebeck et al., 2006; Valentin et al., 2006). However, further research is warranted on tocopherol biosynthesis-related phytol hydrolyzing activities, an area not yet adequately investigated (Zhang et al., 2014).

The plastidal metabolite geranylgeranyl-PP (GGPP) is an intermediate in the phytyl-PP biosynthetic pathway originating from chlorophyll (Chl), and it is active throughout ripening of the olive fruit (Tanaka et al., 1999), further supported from the up-regulation observed in *geranylgeranyl reductase* expression. During the early stage of *me*socarp development, elevated levels of Chl lead to an increased production of phytyl-PP, which in turn fuels the development of tocopherols and tocotrienols. As the olive fruit is changing color and the quantity of Chl is decreasing, the reaction is still active (*geranylgeranyl reductase* is up-regulated) but leads to lower production of phytyl-PP and, hence, lower production of tocopherols and tocotrienols. After the olive fruit becomes completely black and Chl degrades completely, a transient increase in expression levels geranylgeranyl reductase was observed, signaling a potential involvement of the specific metabolite in a different pathway. Overall, the up-regulation levels of geranylgeranyl reductase in green olive fruit were lower than those of the black olive fruit, in agreement with previous reports (Bruno et al., 2009; Muzzalupo et al., 2011).

As far as the intermediate genes in the biosynthetic pathway are concerned, *HGGT and VTE2* were both suppressed in all weeks after flowering. It should be noted that *VTE2* and *HGGT* are involved in tocopherol and tocotrienol biosynthesis, respectively. These two genes demonstrated the highest levels of down-regulation at 8, 20, and 32 WAF (Figure 2). Low levels of all tocochromanols were also detected at 32 WAF which can be partially justified by the low expression levels of *geranylgeranyl reductase, VTE2, HGGT, VTE3, VTE1*, and *VTE4*. Such low transcript and metabolite levels might be attributed at harvest maturity. The same pattern was observed with highest levels of down-regulation for *VTE2* and *HGGT*, while high levels of all tocochromanols were monitored at 8 WAF. At this stage, the fruit is 50% of its final size and the stone becomes lignified. It is also possible that higher concentrations of all tocochromanols result in a negative feedback regulatory mechanism, thus inhibiting these HPPD, VTE2, and HGGT biosynthetic enzymes. The same negative feedback regulation pattern has been also observed in other biological systems such as that in *M. truncatula* plants where nitric oxide (NO) accumulation results in a decline in nitrate reductase (NR) activity, the major contributor of NO biosynthesis (Antoniou et al., 2013).

It should be pointed out that the characterization of gene expression using a qRT-PCR approach with a single reference gene in a time-course experiment comprising a large number of time points could potentially pose problems, particularly in the case that more than one developmental programmes are involved such as fruit growth and ripening. There is the possibility that slight variations in expression of the reference gene might prevent detection of expression alterations with biological significance. In addition, it might increase the variation of its expression due to the biological replicates which have to be performed. Nevertheless, *Ct* values obtained for *UBQ2* in this setup were very similar across all samples examined (mean *Ct* value 21.15, standard error of 0.12, standard deviation of 0.85).

The highest concentrations of metabolites (tocopherols and tocotrienols) were detected during the first and intermediate stages of olive fruit development, peaking at 22 WAF. Starting with fruit ripening (after 22 WAF), a steady decrease of tocochromanols was detected (Figure 3). Recently, Bodoira et al. (2015) also showed high amounts of tocochromanol content during the early stages of olive fruit (cv. Arauco) development, but observed a decreasing pattern throughout all developmental stages. The concentration of α-tocopherol (16.15 ± 0.60−32.45 ± 0.54 mg/100 g F.W.) in olive fruit is significantly greater than those of the β-, γ-, and δ-tocopherols (0.13 ± 0.01−0.25 ± 0.03 mg/100 g F.W., 0.13 ± 0.01−0.33 ± 0.04 mg/100 g F.W., 0.14 ± 0.01−0.28 ± 0.01 mg/100 g F.W., respectively), similar to the results by Hassapidou and Manoukas (1993), Bruno et al. (2009), Muzzalupo et al. (2011), and Bodoira et al. (2015). Interestingly, the concentrations of γ-tocopherol are only slightly lower than those of α-tocopherol at the early stages of “Arauco” olive fruit (Bodoira et al., 2015). The γ-tocotrienol was detected at low concentrations and α-, β-, and δ-tocotrienols were non-detectable (Figure 4); thus indicating that varietal differences expected to occur in tocochromanol content among olive cultivars. Overall, olive fruit contained significantly higher concentrations of tocopherols and tocotrienols until 22 WAF (concomitant with the end of mesocarp development) compared with later stages, thus suggesting that the color change phase might be of critical importance in vitamin E content of olive fruit.

From all the tocochromanols examined, α-tocopherol was the most abundant with an average percentage of 96.72 ± 0.16% of total concentration in 6–38 WAF, with higher concentrations being detected within the period starting 6 WAF (24.41 ± 1.99 mg/100 g F.W.) and ending at 22 WAF (27.01 ± 1.05 mg/100 g F.W., the period of mesocarp development). α-tocopherol content remained stable and at the lowest concentrations from the color change (“breaker stage”) up to over-ripe phase. The abundance of α-tocopherol in olive fruit is evident in several other studies, where it reaches concentration percentages between 60.9 and 88.6% depending on cultivar and developmental stage considered. The content of the other tocopherols fluctuated between 2.7 and 14.2% β-tocopherol, 0.6–41.8% γ-tocopherol, and 3.8–25.9% δ-tocopherol (Hassapidou and Manoukas, 1993; Bruno et al., 2009; Muzzalupo et al., 2011; Bodoira et al., 2015).

Finally, low concentrations of all tocochromanols were detected at 36 WAF, concomitant with the highest levels of induction in *HPPD* gene expression. Such low concentrations of tocochromanols and up-regulation of *HPPD* might be affected by the over-ripening processes. Even though the levels of *HPPD* increased at 36 WAF, tocochromanol content decreased, probably due to the presence of other enzymes in the pathway (Ren et al., 2011). It is possible that when a metabolite reaches low threshold concentrations, its biosynthesis is subsequently induced at a transcript level. In various plants, where an induction in *HPPD* is observed, such increases normally concur with small to moderate increases in total concentration of vitamin E (Zhang et al., 2013).

## Conclusions

This report is a first attempt for the temporal characterization of the vitamin E biosynthesis in olive fruit during on-tree development and ripening (Figure 5). Current findings suggest that olive fruits have increased amounts of all tocopherols and γ-tocotrienol up to 22 WAF (beginning of color change) in comparison with later WAFs, correlating with the expression profile of *VTE5* which could thus be proposed as a marker gene for vitamin E analyses. Alpha-tocopherol is the predominant tocochromanol, similar to other plant species. Further research on vitamin E biosynthetic enzyme activities and protein abundance (components shown in Figure 1) could provide valuable biochemical evidence toward the complete mapping of the biosynthetic pathway of vitamin E in olive fruit.

### Conflict of interest statement

The authors declare that the research was conducted in the absence of any commercial or financial relationships that could be construed as a potential conflict of interest.

## Abbreviations

WAF: Weeks after flowering
UBQ2: Polyubiquitin2
HPPD: p- or 4-hydroxyphenylpyruvate dioxygenase
HPT or VTE2: homogentisate phytyl transferase or vitamin E2
VTE5: Phytol kinase or vitamin E5
HGGT: Homogentisate geranylgeranyl transferase
MPBQ MT or VTE3: 2-methyl-6-phytyl-1,4-benzoquinol methyl transferase or vitamin E3
TC or VTE1: Tocopherol cyclase or vitamin E1
γ-TMT or VTE4: γ-tocopherol methyl transferase or vitamin E4
phytyl-P or PMP: Phytyl phosphate
phytyl-PP or PDP: Phytyl diphosphate
GGPP or GGDP: geranylgeranyl pyrophosphate or geranylgeranyl diphosphate
HPP: p- or 4-hydroxyphenylpyruvic acid
HGA: Homogentisic acid
MPBQ: 2-methyl-6-phytylbenzoquinol
DMPBQ: 2, 3-dimethyl-6-phytyl-1,4-benzoquinol
MGGBQ: 2-methyl-6-geranylgeranylbenzoquinol
DMGGBQ: 2, 3-dimethyl-6-geranylgeranyl-1,4-benzoquinol
F.W.: fresh weight.
