# Supplementary material for: Temporal analysis reveals a key role for VTE5 in vitamin E biosynthesis in olive fruit during on-tree development
Source: Front Plant Sci. 2015 Oct 21;6:871. doi: 10.3389/fpls.2015.00871 (PMC4617049; doi:10.3389/fpls.2015.00871)
Supplement: Supplementary file 1 [file Tables1-11.doc]

# *Supplementary* *Material*

**Temporal analysis reveals a key role for *VTE5* in vitamin E biosynthesis in olive fruit during on-tree development**

Egli C. Georgiadou, Thessaloniki Ntourou, Vlasios Goulas, George A. Manganaris, Panagiotis Kalaitzis, Vasileios Fotopoulos*

*** Correspondence: Vasileios Fotopoulos:** [vassilis.fotopoulos@cut.ac.cy](mailto:vassilis.fotopoulos@cut.ac.cy)

##

**Supplementary Table 1.** Harvest data on olive fruit of ‘Koroneiki’ cultivar used in this study.

| **Developmental stages** | **Harvest Dates** | **Weeks After Flowering**  **(WAF)** |
| --- | --- | --- |
| *S_1_* | 14 June 2006 | 6 |
| *S_2_* | 30 June 2006 | 8 |
| *S_3_* | 12 July 2006 | 10 |
| *S_4_* | 26 July 2006 | 12 |
| *S_5_* | 9 August 2006 | 14 |
| *S_6_* | 23 August 2006 | 16 |
| *S_7_* | 6 September 2006 | 18 |
| *S_8_* | 20 September 2006 | 20 |
| *S_9_* | 4 October 2006 | 22 |
| *S_10_* | 18 October 2006 | 24 |
| *S_11_* | 1 November 2006 | 26 |
| *S_12_* | 15 November 2006 | 28 |
| *S_13_* | 29 November 2006 | 30 |
| *S_14_* | 13 December 2006 | 32 |
| *S_15_* | 27 December 2006 | 34 |
| *S_16_* | 10 January 2006 | 36 |
| *S_17_* | 24 January 2006 | 38 |

**Supplementary Table 2.** Phenological growth stages of olive trees used in this study based on the BBCH (Biologische Bundesanstalt, Bundessortenamt, Chemische Industrie) scale. The mesocarp developmental phase corresponds to 6-22 WAF, while the ripening phase of the olive fruit corresponds to 22-38 WAF.

| **Develop-mental stages** | **Phenological growth stage** | | **Weeks after flowering (WAF)** | | | | | | | | | | | | | | | | |
| --- | --- | --- | --- | --- | --- | --- | --- | --- | --- | --- | --- | --- | --- | --- | --- | --- | --- | --- | --- |
|  |  |  | 6 | 8 | 10 | 12 | 14 | 16 | 18 | 20 | 22 | 24 | 26 | 28 | 30 | 32 | 34 | 36 | 38 |
| ***S_1_*** | 71 | Fruit about 10% of final size |  |  |  |  |  |  |  |  |  |  |  |  |  |  |  |  |  |
| ***S_2_ – S_6_*** | 75 | Fruit about 50% of final size, stone becomes lignified |  |  |  |  |  |  |  |  |  |  |  |  |  |  |  |  |  |
| ***S_7_* – *S_8_*** | 79 | Fruit about 90% of final size, fruit suitable for picking green |  |  |  |  |  |  |  |  |  |  |  |  |  |  |  |  |  |
| ***S_9_* – *S_10_*** | 81 | Beginning of fruit colouring |  |  |  |  |  |  |  |  |  |  |  |  |  |  |  |  |  |
| ***S_11_*** | 85 | Increasing specific fruit colouring |  |  |  |  |  |  |  |  |  |  |  |  |  |  |  |  |  |
| ***S_12_ – S_15_*** | 89 | Harvest maturity |  |  |  |  |  |  |  |  |  |  |  |  |  |  |  |  |  |
| ***S_16_ - S_17_*** | 92 | Over-ripe |  |  |  |  |  |  |  |  |  |  |  |  |  |  |  |  |  |
|  | | | **🡨mesocarp development🡪** | | | | | | | | | **🡨 ripening 🡪** | | | | | | | |

**Supplementary Table 3.** Accession numbers corresponding ESTs from OLEA EST db (<http://140.164.45.140/oleaestdb/search.php>).

| **Gene** | **Accession number** | Expressed sequence tag (ESTs) |
| --- | --- | --- |
| *VTE5* | E8NTSAO03HA0AH | GGAAACTGGTCCACATATTGTCGGGATTGCTTTTCTTTGGCTGCCTGGCCAATT  TTCAGGTACCTGCTTTGTCGTTGGAGAGTTACAGTCTGCATTTCTGTTAATTAA  TCATTATGTTATCAAAAACTTCAACTGAATATTTTGATCTTAGATAGCACATCGA  CAGGAGCTCGCTACTTTGCTTCTTTAGTTCCTCTTATAAATTGTTTAAGGCTTTT  GATACATGGTCTTTCCTTGGCTACTGATGAAGGACTCGTCAAA |
| *HPPD* | E8NTSAO02CY14E | ATATATCCTCAGATACCAATGCCAAATGCTGCACCCCTGCCCCTTCATTGTGCT  CCAAGTAAGTCTGTATCTGGCTCTTCCTTTTAGTTCCATGTACAGGTTCATTCA  AAGGCAACAACACAGTCTCGTCGTTATTAGCTAGAACAACCGAGTTCAGTCC  GCTATCGGCCGTCCCCACATCCTCAGTCGTAAATTCTGCAAATTCATGGAATC  CAGTGGACGTTTTTATGTACTCAACACAGGCCG |
| *VTE2* | E8NTSAO01BSBQ9 | AATAGTGAAAGAATAGTTGCATTGAGTCCAAGATGCAGATCAAAGTATCTAG  TGAGTGCAACTTCTGAACACAATCTTGAATCCGAACCTTCCAAGAGCCGTGG  GAGATCAATTCAACATGCATTAGATGCTTTCTACAGGTTTTCACGGCCCCACA  CAGTAATAGGAACAGCATTGAGCATAATTTCAGTTTCTCTCCTTGCAGTAGAA  AATCTCTCCGATATTTCTCCATTATTTGT |
| *HGGT* | E8NTSAO04IZCRI | GTGACTGAATATCCTCCCGATGCCAATGGAAGATATGGCTTGTTTACCTTATCT  ATTTCAATGTCAGACAGCTGATTCAAACCGACTATGTATATGTTCATCAAAAG  GGCCGCAGCAATGGCCTCTATCACCCCAGTGACAAATAATGGAGAAATATCG  GAGAGATTTTCTACTGCAAGGAGAGAAACTGAAATTATGCTCAATGCTGTTC  CTATTACTGTGTGGGGCCGTGAAAACCTGTAGAAAG |
| *VTE3* | E8NTSAO01BS0YR | CGAGCATCCCATAATCAGCCCATGTCTACGAACCCCACGATACCATTTTGGAC  CAATCCTCTTCAGTTGAACATCCTTGAATCCAGCCTTTTCAAACCACTCAATG  TACTCTTCCTCCTTCGGGAAGAGCATCCACACATCAGCAAAGAAGCGAGATA  ACCAAAATGTTGGATATACAGGACCAATTACGCAAGCCTTCCCTCCGAGCCTT  AAAACTCGATATGCTTCCCTGATGCCACGCTGTGGGTCTGG |
| *VTE1* | E8NTSAO03HE119 | GAAAAACGATATGATGGCAGTGAAGGAAAGGTTATACTTGATGTGACAAGT  AGCATGGCAGCCGTAGAAGTTGGAGGAGGACCATGGTTCAACACATGGAA  AGGCAAGACATATACACCGGAAACTATAAAACGAGCTCTTACGCTTCCTGTT  GATGTGGAAGGGATAGTTGGTTTGGTTCCTTTCCTTAAACCTCCTGGCTTGTA  GATGATTTGGATTAATGCACACCTGCAGCACCATAAATGAATCAAGAGGTAG |
| *VTE4* | E8NTSAO03GW1S2 | AAGAGCTGTTAAACAAGATATGTAATGCTTTTTACCTTCCGGCATGGTGTTCT  ACTGCTGATTATGTAAAATTACTTGAATCCCTCTCTCTTCAGGATATTAAAGCT  GCAGACTAGGTCTGAATATGTTGCCCCATTTTGGCCAGCAGTTATAAAGTCAG  CATTGACATGGAAGGGAATCACCTCATTGTTAAGAAGTGGATGGAAGACAATA  AAAGGAGCACTGGCCATGCCATTGATGATCGG |

**Supplementary Table 4.** Oligonucleotide primers used for real-time RT-PCR analyses. Accession numbers with * are from NCBI database (http://www.ncbi.nlm.nih.gov/), while others are ESTs from OLEA EST db (<http://140.164.45.140/oleaestdb/search.php>).

| **Gene** | **Accession number** | **Primer** | **Nucleotide sequence 5’-3’** | **Τm (°C)** |
| --- | --- | --- | --- | --- |
| *UBQ2* | AF429430^*^ | *UBQ2*-For: | AATGAAGTCTGTCTCTCCTTTGG | 56 |
|  |  | *UBQ2*-Rev: | AAGGGAAATCCCATCAACG |  |
| *VTE5* | E8NTSAO03HA0AH | *VTE5*-For: | CTGCTTTGTCGTTGGAGAGA | 65 |
|  |  | *VTE5*-Rev: | TCAGTAGCCAAGGAAAGACCA |  |
| *Geranylgeranyl*  *reductase* | DQ424963^*^ | *GGR* -For: | CCAAGGGAGGCATTTGTAGA | 54 |
|  |  | *GGR* -Rev: | TGGATTCACAGCCAATTTCA |  |
| *HPPD* | E8NTSAO02CY14E | *HPPD*-For: | CCCTTCATTGTGCTCCAAGT | 54 |
|  |  | *HPPD*-Rev: | CCACTGGATTCCATGAATTTG |  |
| *VTE2* | E8NTSAO01BSBQ9 | *VTE2*-For: | TTGCATTGAGTCCAAGATGC | 54 |
|  |  | *VTE2*-Rev: | GAAATTATGCTCAATGCTGTTCC |  |
| *HGGT* | E8NTSAO04IZCRI | *HGGT*-For: | AATATCCTCCCGATGCCAAT | 54 |
|  |  | *HGGT*-Rev: | ACGGCCCCACACAGTAATAG |  |
| *VTE3* | E8NTSAO01BS0YR | *VTE3*-For: | CCCACGATACCATTTTGGAC | 54 |
|  |  | *VTE3*-Rev: | GCTTGCGTAATTGGTCCTGT |  |
| *VTE1* | E8NTSAO03HE119 | *VTE1*-For: | TCAACACATGGAAAGGCAAG | 65 |
|  |  | *VTE1*-Rev: | CATTTATGGTGCTGCAGGTG |  |
| *VTE4* | E8NTSAO03GW1S2 | *VTE4*-For: | TTCCGGCATGGTGTTCTACT | 54 |
|  |  | *VTE4*-Rev: | GGCCAGTGCTCCTTTTATTG |  |

**Supplementary Table 5.** Protein homology of VTE5 in olives involved in the biosynthetic pathway of vitamin E.

| **Gene** | **Accession Number** | **Plant Species** | **Percentage**  **Identity** | **Percentage**  **Similarity** | **E-value** |
| --- | --- | --- | --- | --- | --- |
| *VTE5* | [XP_002269950.](http://www.ncbi.nlm.nih.gov/protein/225448861?report=genbank&log$=prottop&blast_rank=2&RID=X36T70NY014)1 | *Vitis vinifera* | 83 % | 83 % | 1e-11 |
|  | [XP_010665628.1](http://www.ncbi.nlm.nih.gov/protein/731370145?report=genbank&log$=prottop&blast_rank=4&RID=X36T70NY014) | *Beta vulgaris subsp. vulgaris* | 71 % | 97 % | 3e-11 |
|  | [XP_008218941.1](http://www.ncbi.nlm.nih.gov/protein/645224089?report=genbank&log$=prottop&blast_rank=5&RID=X36T70NY014) | *Prunus mume* | 81 % | 83 % | 3e-11 |
|  | [XP_008375637.1](http://www.ncbi.nlm.nih.gov/protein/657967897?report=genbank&log$=prottop&blast_rank=8&RID=X36T70NY014) | *Malus domestica* | 81 % | 83 % | 1e-10 |
|  | [XP_009591844.1](http://www.ncbi.nlm.nih.gov/protein/697166062?report=genbank&log$=prottop&blast_rank=15&RID=X36T70NY014) | *Nicotiana tomentosiformis* | 81 % | 83 % | 7e-10 |
|  | XP_011024295.1 | *Populus euphratica* | 75 % | 88 % | 2e-10 |
|  | [EEF29465](http://www.ncbi.nlm.nih.gov/protein/255584378?report=genbank&log$=prottop&blast_rank=17&RID=X36T70NY014).1 | *Ricinus communis* | 75 % | 83 % | 1e-09 |
|  | [XP_011094481.1](http://www.ncbi.nlm.nih.gov/protein/747093358?report=genbank&log$=prottop&blast_rank=19&RID=X36T70NY014) | *Sesamum indicum* | 75 % | 83 % | 2e-09 |
|  | [KDP45055](http://www.ncbi.nlm.nih.gov/protein/802547436?report=genbank&log$=prottop&blast_rank=21&RID=X36T70NY014).1 | *Jatropha curcas* | 78 % | 83 % | 3e-09 |
|  | [XP_006588440.1](http://www.ncbi.nlm.nih.gov/protein/571480811?report=genbank&log$=prottop&blast_rank=22&RID=X36T70NY014) | *Glycine max* | 72 % | 83 % | 3e-09 |
|  | [XP_006364441.1](http://www.ncbi.nlm.nih.gov/protein/565397736?report=genbank&log$=prottop&blast_rank=24&RID=X36T70NY014) | *Solanum tuberosum* | 81 % | 83 % | 4e-09 |
|  | [XP_009783010.1](http://www.ncbi.nlm.nih.gov/protein/698466854?report=genbank&log$=prottop&blast_rank=30&RID=X36T70NY014) | *Nicotiana sylvestris* | 78 % | 83 % | 7e-09 |
|  | [ESW16295](http://www.ncbi.nlm.nih.gov/protein/593687283?report=genbank&log$=prottop&blast_rank=32&RID=X36T70NY014).1 | *Phaseolus vulgaris* | 72 % | 83 % | 8e-09 |
|  | [KDO55302](http://www.ncbi.nlm.nih.gov/protein/567888122?report=genbank&log$=prottop&blast_rank=34&RID=X36T70NY014).1 | *Citrus sinensis* | 73 % | 86 % | 1e-08 |
|  | [XP_004234914.1](http://www.ncbi.nlm.nih.gov/protein/460378306?report=genbank&log$=prottop&blast_rank=37&RID=X36T70NY014) | *Solanum lycopersicum* | 81 % | 83 % | 2e-08 |
|  | [XP_010067215.1](http://www.ncbi.nlm.nih.gov/protein/702423130?report=genbank&log$=prottop&blast_rank=42&RID=X36T70NY014) | *Eucalyptus grandis* | 75 % | 83 % | 4e-08 |
|  | [XP_006653001.1](http://www.ncbi.nlm.nih.gov/protein/573941213?report=genbank&log$=prottop&blast_rank=40&RID=X36T70NY014) | *Oryza brachyantha* | 66 % | 88 % | 4e-08 |
|  | [ABA42672.1](http://www.ncbi.nlm.nih.gov/protein/76443929?report=genbank&log$=prottop&blast_rank=60&RID=X36T70NY014) | *Zea mays* | 66 % | 88 % | 9e-07 |
|  | EOY18924.1 | *Theobroma cacao* | 69 % | 83 % | 8e-07 |
|  | [XP_004496158.1](http://www.ncbi.nlm.nih.gov/protein/502118218?report=genbank&log$=prottop&blast_rank=62&RID=X36T70NY014) | *Cicer arietinum* | 64 % | 83 % | 1e-06 |
|  | [KEH43364.1](http://www.ncbi.nlm.nih.gov/protein/657404741?report=genbank&log$=prottop&blast_rank=63&RID=X36T70NY014) | *Medicago truncatula* | 67 % | 83 % | 3e-06 |
|  | [XP_009396489.1](http://www.ncbi.nlm.nih.gov/protein/694996874?report=genbank&log$=prottop&blast_rank=65&RID=X36T70NY014) | *Musa acuminata subsp. malaccensis* | 67 % | 83 % | 3e-06 |
|  | [XP_008795886.1](http://www.ncbi.nlm.nih.gov/protein/672143993?report=genbank&log$=prottop&blast_rank=69&RID=X36T70NY014) | *Phoenix dactylifera* | 58 % | 83 % | 1e-05 |
|  | [CDX70220.1](http://www.ncbi.nlm.nih.gov/protein/685379801?report=genbank&log$=prottop&blast_rank=76&RID=X36T70NY014) | *Brassica napus* | 56 % | 83 % | 5e-05 |
|  | ABA42674.1 | *Triticum aestivum* | 58 % | 88 % | 9e-05 |
|  | AED90752.1 | *Arabidopsis thaliana* | 58 % | 88 % | 6e-04 |
|  | [XP_010452347.1](http://www.ncbi.nlm.nih.gov/protein/727560253?report=genbank&log$=prottop&blast_rank=88&RID=X36T70NY014) | *Camelina sativa* | 56 % | 83 % | 0.001 |
|  | AFB74217.1 | *Brassica napus* | 53 % | 83 % | 0.001 |
|  | [XP_003580789.1](http://www.ncbi.nlm.nih.gov/protein/357166664?report=genbank&log$=prottop&blast_rank=90&RID=X36T70NY014) | *Brachypodium distachyon* | 53 % | 88 % | 0.002 |
|  | [XP_004307151.1](http://www.ncbi.nlm.nih.gov/protein/470142948?report=genbank&log$=prottop&blast_rank=102&RID=X36T70NY014) | *Fragaria vesca subsp. vesca* | 72 % | 83 % | 0.66 |

**Supplementary Table 6.** Protein homology of HPPD in olives involved in the biosynthetic pathway of vitamin E.

| **Gene** | **Accession Number** | **Plant Species** | **Percentage**  **Identity** | **Percentage**  **Similarity** | **E-value** |
| --- | --- | --- | --- | --- | --- |
| *HPPD* | [ADZ24701.1](http://www.ncbi.nlm.nih.gov/protein/325516246?report=genbank&log$=prottop&blast_rank=1&RID=WMHBY58D01R) | *Solanum pennellii* | 96 % | 100 % | 9e-26 |
|  | [XP_010087866.1](http://www.ncbi.nlm.nih.gov/protein/703067047?report=genbank&log$=prottop&blast_rank=5&RID=WMHBY58D01R) | *Morus notabilis* | 92 % | 100 % | 5e-25 |
|  | [AFB74208.1](http://www.ncbi.nlm.nih.gov/protein/377657549?report=genbank&log$=prottop&blast_rank=9&RID=WMHBY58D01R) | *Brassica napus* | 94 % | 100 % | 7e-25 |
|  | [ADZ24700.1](http://www.ncbi.nlm.nih.gov/protein/325516244?report=genbank&log$=prottop&blast_rank=11&RID=WMHBY58D01R) | *Solanum pennellii* | 94 % | 100 % | 8e-25 |
|  | [XP_002892349.1](http://www.ncbi.nlm.nih.gov/protein/297848936?report=genbank&log$=prottop&blast_rank=19&RID=WMHBY58D01R) | *Arabidopsis lyrata subsp. lyrata* | 90 % | 100 % | 3e-24 |
|  | [NP_001235148.1](http://www.ncbi.nlm.nih.gov/protein/351721017?report=genbank&log$=prottop&blast_rank=22&RID=WMHBY58D01R) | *Glycine max* | 90 % | 100 % | 6e-24 |
|  | [AIN39549.1](http://www.ncbi.nlm.nih.gov/protein/683418238?report=genbank&log$=prottop&blast_rank=23&RID=WMHBY58D01R) | *Glycine max* | 90 % | 100 % | 6e-24 |
|  | [AFB74207.1](http://www.ncbi.nlm.nih.gov/protein/377657547?report=genbank&log$=prottop&blast_rank=25&RID=WMHBY58D01R) | *[Brassica napus](http://blast.ncbi.nlm.nih.gov/Blast.cgi#alnHdr_377657547)* | 88 % | 100 % | 8e-24 |
|  | [AAC62457.1](http://www.ncbi.nlm.nih.gov/protein/3694811?report=genbank&log$=prottop&blast_rank=28&RID=WMHBY58D01R) | *[Arabidopsis thaliana](http://blast.ncbi.nlm.nih.gov/Blast.cgi#alnHdr_3694811)* | 88 % | 100 % | 9e-24 |
|  | [ADO62712.1](http://www.ncbi.nlm.nih.gov/protein/309260073?report=genbank&log$=prottop&blast_rank=36&RID=WMHBY58D01R) | *Mangifera indica* | 92 % | 100 % | 1e-23 |
|  | [NP_001154311.1](http://www.ncbi.nlm.nih.gov/protein/238478369?report=genbank&log$=prottop&blast_rank=41&RID=WMHBY58D01R) | *Arabidopsis thaliana* | 88 % | 100 % | 2e-23 |
|  | [AFB74218.1](http://www.ncbi.nlm.nih.gov/protein/377657569?report=genbank&log$=prottop&blast_rank=46&RID=WMHBY58D01R) | *Brassica napus* | 88 % | 100 % | 2e-23 |
|  | [AGG10562.1](http://www.ncbi.nlm.nih.gov/protein/452215768?report=genbank&log$=prottop&blast_rank=53&RID=WMHBY58D01R) | *Amaranthus tuberculatus* | 88 % | 100 % | 3e-23 |
|  | [XP_002300867.2](http://www.ncbi.nlm.nih.gov/protein/566156450?report=genbank&log$=prottop&blast_rank=56&RID=WMHBY58D01R) | *[Populus trichocarpa](http://blast.ncbi.nlm.nih.gov/Blast.cgi#alnHdr_566156450)* | 90 % | 100 % | 3e-23 |
|  | [NP_172144.2](http://www.ncbi.nlm.nih.gov/protein/30679736?report=genbank&log$=prottop&blast_rank=59&RID=WMHBY58D01R) | *Arabidopsis thaliana* | 88 % | 100 % | 3e-23 |
|  | [AAM96960.1](http://www.ncbi.nlm.nih.gov/protein/22530912?report=genbank&log$=prottop&blast_rank=60&RID=WMHBY58D01R) | *Arabidopsis thaliana* | 88 % | 100 % | 3e-23 |
|  | [BAH10638.1](http://www.ncbi.nlm.nih.gov/protein/219842162?report=genbank&log$=prottop&blast_rank=63&RID=WMHBY58D01R) | *Hevea brasiliensis* | 90 % | 100 % | 4e-23 |
|  | [XP_003617391.1](http://www.ncbi.nlm.nih.gov/protein/357494205?report=genbank&log$=prottop&blast_rank=76&RID=WMHBY58D01R) | *Medicago truncatula* | 87 % | 100 % | 2e-22 |
|  | [AAN28922.1](http://www.ncbi.nlm.nih.gov/protein/23505684?report=genbank&log$=prottop&blast_rank=81&RID=WMHBY58D01R) | *Abutilon theophrasti* | 83 % | 100 % | 9e-22 |
|  | [ACN78586.1](http://www.ncbi.nlm.nih.gov/protein/225001452?report=genbank&log$=prottop&blast_rank=87&RID=WMHBY58D01R) | *Lactuca sativa* | 87 % | 100 % | 2e-21 |

**Supplementary Table 7.** Protein homology of VTE2 in olives involved in the biosynthetic pathway of vitamin E.

| **Gene** | **Accession Number** | **Plant Species** | **Percentage**  **Identity** | **Percentage**  **Similarity** | **E-value** |
| --- | --- | --- | --- | --- | --- |
| *VTE2* | XP_011069933.1 | *Sesamum indicum* | 70 % | 98 % | 2e-24 |
|  | [ADZ24707.1](http://www.ncbi.nlm.nih.gov/protein/325516258?report=genbank&log$=prottop&blast_rank=12&RID=WMNMFK85015) | *Solanum* *pennellii* | 70 % | 95 % | 4e-23 |
|  | [NP_001241496.1](http://www.ncbi.nlm.nih.gov/protein/359806410?report=genbank&log$=prottop&blast_rank=20&RID=WMNMFK85015) | *Glycine max* | 68 % | 95 % | 2e-22 |
|  | [NP_001238372.1](http://www.ncbi.nlm.nih.gov/protein/351725439?report=genbank&log$=prottop&blast_rank=26&RID=WMNMFK85015) | *Glycine max* | 67 % | 95 % | 8e-22 |
|  | [ACC86836.1](http://www.ncbi.nlm.nih.gov/protein/186695349?report=genbank&log$=prottop&blast_rank=28&RID=WMNMFK85015) | *Coriandrum sativum* | 70 % | 95 % | 2e-21 |
|  | [KHN17607.1](http://www.ncbi.nlm.nih.gov/protein/734365228?report=genbank&log$=prottop&blast_rank=36&RID=WMNMFK85015) | *Glycine soja* | 75 % | 83 % | 6e-20 |
|  | [ACC77744.1](http://www.ncbi.nlm.nih.gov/protein/185487419?report=genbank&log$=prottop&blast_rank=37&RID=WMNMFK85015) | *Manihot esculenta* | 71 % | 91 % | 6e-20 |
|  | [XP_010094206.1](http://www.ncbi.nlm.nih.gov/protein/703090892?report=genbank&log$=prottop&blast_rank=40&RID=WMNMFK85015) | *Morus notabilis* | 66 % | 95 % | 1e-19 |
|  | [AES92284.2](http://www.ncbi.nlm.nih.gov/protein/657391238?report=genbank&log$=prottop&blast_rank=54&RID=WMNMFK85015) | *Medicago truncatula* | 63 % | 95 % | 2e-18 |
|  | [XP_003610087.1](http://www.ncbi.nlm.nih.gov/protein/357479603?report=genbank&log$=prottop&blast_rank=58&RID=WMNMFK85015) | *Medicago truncatula* | 63 % | 95 % | 3e-18 |
|  | [AHL26475.1](http://www.ncbi.nlm.nih.gov/protein/590001296?report=genbank&log$=prottop&blast_rank=70&RID=WMNMFK85015) | *Elaeis oleifera* | 64 % | 86 % | 2e-17 |
|  | [NP_001105877.1](http://www.ncbi.nlm.nih.gov/protein/162461713?report=genbank&log$=prottop&blast_rank=81&RID=WMNMFK85015) | *Zea mays* | 69 % | 80 % | 1e-16 |
|  | [BAH10640.1](http://www.ncbi.nlm.nih.gov/protein/219842166?report=genbank&log$=prottop&blast_rank=88&RID=WMNMFK85015) | *[Hevea brasiliensis](http://blast.ncbi.nlm.nih.gov/Blast.cgi#alnHdr_219842166)* | 66 % | 88 % | 2e-16 |
|  | [ADJ21814.1](http://www.ncbi.nlm.nih.gov/protein/299507806?report=genbank&log$=prottop&blast_rank=91&RID=WMNMFK85015) | *Solanum tuberosum* | 59 % | 98 % | 3e-16 |
|  | [NP_849984.1](http://www.ncbi.nlm.nih.gov/protein/30680535?report=genbank&log$=prottop&blast_rank=92&RID=WMNMFK85015) | *Arabidopsis thaliana* | 59 % | 98 % | 3e-16 |
|  | [KMZ67650.1](http://www.ncbi.nlm.nih.gov/protein/901813159?report=genbank&log$=prottop&blast_rank=99&RID=WMNMFK85015) | *Zostera marina* | 73 % | 76 % | 6e-16 |
|  | [ABB70124.1](http://www.ncbi.nlm.nih.gov/protein/81295662?report=genbank&log$=prottop&blast_rank=108&RID=WPX1YKSH01R) | *Allium ampeloprasum* | 62 % | 86 % | 1e-14 |
|  | [ABB70123.1](http://www.ncbi.nlm.nih.gov/protein/81295660?report=genbank&log$=prottop&blast_rank=109&RID=WPX1YKSH01R) | *Triticum aestivum* | 72 % | 73 % | 1e-14 |
|  | [AFB74212.1](http://www.ncbi.nlm.nih.gov/protein/377657557?report=genbank&log$=prottop&blast_rank=112&RID=WPX1YKSH01R) | *Brassica napus* | 58 % | 98 % | 1e-14 |
|  | [AFB74211.1](http://www.ncbi.nlm.nih.gov/protein/377657555?report=genbank&log$=prottop&blast_rank=114&RID=WPX1YKSH01R) | *Brassica napus* | 56 % | 98 % | 3e-14 |
|  | [ACN81039.1](http://www.ncbi.nlm.nih.gov/protein/225125594?report=genbank&log$=prottop&blast_rank=128&RID=WPX1YKSH01R) | *Linum usitatissimum* | 91 % | 47 % | 3e-11 |

**Supplementary Table 8.** Protein homology of HGGT in olives involved in the biosynthetic pathway of vitamin E.

| **Gene** | **Accession Number** | **Plant Species** | **Percentage**  **Identity** | **Percentage**  **Similarity** | **E-value** |
| --- | --- | --- | --- | --- | --- |
| *HGGT* | [XP_007014228.1](http://www.ncbi.nlm.nih.gov/protein/590581005?report=genbank&log$=prottop&blast_rank=46&RID=WMXVXFZF01R) | *Theobroma cacao* | 81 % | 100 % | 8e-28 |
|  | [XP_007014227.1](http://www.ncbi.nlm.nih.gov/protein/590581001?report=genbank&log$=prottop&blast_rank=47&RID=WMXVXFZF01R) | *Theobroma cacao* | 81 % | 100 % | 8e-28 |
|  | [XP_010656795.1](http://www.ncbi.nlm.nih.gov/protein/731408264?report=genbank&log$=prottop&blast_rank=50&RID=WMXVXFZF01R) | *Vitis vinifera* | 88 % | 100 % | 8e-28 |
|  | [NP_001267827.1](http://www.ncbi.nlm.nih.gov/protein/526117663?report=genbank&log$=prottop&blast_rank=59&RID=WMXVXFZF01R) | *Vitis vinifera* | 88 % | 100 % | 1e-27 |
|  | [ACG45339.1](http://www.ncbi.nlm.nih.gov/protein/195651743?report=genbank&log$=prottop&blast_rank=103&RID=WPVD5GA701R) | *Zea mays* | 79 % | 100 % | 3e-25 |
|  | [BAH10642.1](http://www.ncbi.nlm.nih.gov/protein/219842170?report=genbank&log$=prottop&blast_rank=137&RID=WPVD5GA701R) | *Hevea brasiliensis* | 61 % | 100 % | 4e-19 |
|  | [XP_004965733.1](http://www.ncbi.nlm.nih.gov/protein/514765361?report=genbank&log$=prottop&blast_rank=148&RID=WPVD5GA701R) | *Setaria italica* | 66 % | 100 % | 2e-18 |
|  | [XP_004965732.1](http://www.ncbi.nlm.nih.gov/protein/514765357?report=genbank&log$=prottop&blast_rank=152&RID=WPVD5GA701R) | *Setaria italica* | 66 % | 100 % | 3e-18 |
|  | [XP_010932231.1](http://www.ncbi.nlm.nih.gov/protein/743822262?report=genbank&log$=prottop&blast_rank=155&RID=WPVD5GA701R) | *Elais guineensis* | 61 % | 100 % | 6e-18 |
|  | [XP_011459931.1](http://www.ncbi.nlm.nih.gov/protein/764549264?report=genbank&log$=prottop&blast_rank=161&RID=WPVD5GA701R) | Fragaria vesca subsp. vesca | 60 % | 97 % | 9e-18 |
|  | [XP_002282953.2](http://www.ncbi.nlm.nih.gov/protein/359476155?report=genbank&log$=prottop&blast_rank=175&RID=WPVD5GA701R) | *Vitis vinifera* | 60 % | 100 % | 2e-17 |
|  | [XP_010324868.1](http://www.ncbi.nlm.nih.gov/protein/723721064?report=genbank&log$=prottop&blast_rank=194&RID=WPVD5GA701R) | *Solanum lycopersicum* | 61 % | 91 % | 4e-16 |
|  | [XP_010227356.1](http://www.ncbi.nlm.nih.gov/protein/721697594?report=genbank&log$=prottop&blast_rank=198&RID=WPVD5GA701R) | *Brachypodium distachyon* | 61 % | 100 % | 4e-16 |
|  | [XP_010324867.1](http://www.ncbi.nlm.nih.gov/protein/723721061?report=genbank&log$=prottop&blast_rank=202&RID=WPVD5GA701R) | *Solanum lycopersicum* | 61 % | 91 % | 6e-16 |
|  | [XP_010265682.1](http://www.ncbi.nlm.nih.gov/protein/720031023?report=genbank&log$=prottop&blast_rank=205&RID=WPVD5GA701R) | *Nelumbonucifera* | 58 % | 100 % | 9e-16 |
|  | [ADG26667.1](http://www.ncbi.nlm.nih.gov/protein/295656253?report=genbank&log$=prottop&blast_rank=216&RID=WPVD5GA701R) | *Coriandrum sativum* | 60 % | 94 % | 2e-15 |
|  | [XP_010324866.1](http://www.ncbi.nlm.nih.gov/protein/723721058?report=genbank&log$=prottop&blast_rank=228&RID=WPVD5GA701R) | *Solanum lycopersicum* | 57 % | 100 % | 2e-14 |
|  | [ACB42448.1](http://www.ncbi.nlm.nih.gov/protein/171190284?report=genbank&log$=prottop&blast_rank=229&RID=WPVD5GA701R) | *Angelica gigas* | 55 % | 97 % | 2e-14 |
|  | [XP_010667279.1](http://www.ncbi.nlm.nih.gov/protein/731317024?report=genbank&log$=prottop&blast_rank=336&RID=WPVD5GA701R) | *Beta vulgaris subsp.vulgaris* | 63 % | 61 % | 4e-09 |

**Supplementary Table 9.** Protein homology of VTE3 in olives involved in the biosynthetic pathway of vitamin E.

| **Gene** | **Accession Number** | **Plant Species** | **Percentage**  **Identity** | **Percentage**  **Similarity** | **E-value** |
| --- | --- | --- | --- | --- | --- |
| *VTE3* | [AEN74939.1](http://www.ncbi.nlm.nih.gov/protein/345114370?report=genbank&log$=prottop&blast_rank=1&RID=WN2X6G9D01R) | *Arachis hypogaea* | 98 % | 100 % | 2e-23 |
|  | [AEN74936.1](http://www.ncbi.nlm.nih.gov/protein/345114364?report=genbank&log$=prottop&blast_rank=2&RID=WN2X6G9D01R) | *Arachis hypogaea* | 98 % | 100 % | 2e-23 |
|  | [ADZ24708.1](http://www.ncbi.nlm.nih.gov/protein/325516260?report=genbank&log$=prottop&blast_rank=5&RID=WN2X6G9D01R) | *Solanum pennellii* | 95 % | 100 % | 5e-23 |
|  | [BAH10641.1](http://www.ncbi.nlm.nih.gov/protein/219842168?report=genbank&log$=prottop&blast_rank=11&RID=WN2X6G9D01R) | *Hevea brasiliensis* | 100 % | 100 % | 5e-22 |
|  | [AEN74937.1](http://www.ncbi.nlm.nih.gov/protein/345114366?report=genbank&log$=prottop&blast_rank=23&RID=WN2X6G9D01R) | *Arachis hypogaea* | 98 % | 100 % | 3e-22 |
|  | [AEN74938.1](http://www.ncbi.nlm.nih.gov/protein/345114368?report=genbank&log$=prottop&blast_rank=27&RID=WN2X6G9D01R) | *Arachis hypogaea* | 98 % | 100 % | 3e-22 |
|  | [BAL46505.1](http://www.ncbi.nlm.nih.gov/protein/373938267?report=genbank&log$=prottop&blast_rank=49&RID=WN2X6G9D01R) | *Diospyros kaki* | 98 % | 100 % | 7e-22 |
|  | [AEU11048.1](http://www.ncbi.nlm.nih.gov/protein/358440847?report=genbank&log$=prottop&blast_rank=50&RID=WN2X6G9D01R) | *Linum usitatissimum* | 98 % | 100 % | 7e-22 |
|  | [ABB52804.1](http://www.ncbi.nlm.nih.gov/protein/80971664?report=genbank&log$=prottop&blast_rank=58&RID=WN2X6G9D01R) | *Helianthus annuus* | 95 % | 100 % | 1e-21 |
|  | [ABB52806.1](http://www.ncbi.nlm.nih.gov/protein/80971668?report=genbank&log$=prottop&blast_rank=60&RID=WN2X6G9D01R) | *Helianthus annuus* | 95 % | 100 % | 1e-21 |
|  | [ACD03289.1](http://www.ncbi.nlm.nih.gov/protein/187373137?report=genbank&log$=prottop&blast_rank=78&RID=WN2X6G9D01R) | *Brassica napus* | 95 % | 100 % | 2e-21 |
|  | [NP_191900.1](http://www.ncbi.nlm.nih.gov/protein/15229430?report=genbank&log$=prottop&blast_rank=80&RID=WN2X6G9D01R) | *Arabidopsis thaliana* | 95 % | 100 % | 2e-21 |
|  | [AFB74214.1](http://www.ncbi.nlm.nih.gov/protein/377657561?report=genbank&log$=prottop&blast_rank=81&RID=WPYDZ7VS01R) | *Brassica napus* | 95 % | 100 % | 2e-21 |
|  | [KEH42806.1](http://www.ncbi.nlm.nih.gov/protein/657404034?report=genbank&log$=prottop&blast_rank=85&RID=WN2X6G9D01R) | *Medicago truncatula* | 95 % | 100 % | 2e-21 |
|  | [EPS61144.1](http://www.ncbi.nlm.nih.gov/protein/527189523?report=genbank&log$=prottop&blast_rank=94&RID=WN2X6G9D01R) | *Genlisea aurea* | 95 % | 100 % | 3e-21 |
|  | [CAX36917.1](http://www.ncbi.nlm.nih.gov/protein/231274771?report=genbank&log$=prottop&blast_rank=103&RID=WPXS5D2001R) | *Triticum aestivum* | 95 % | 100 % | 4e-21 |
|  | [ACP43457.1](http://www.ncbi.nlm.nih.gov/protein/227955444?report=genbank&log$=prottop&blast_rank=105&RID=WPXS5D2001R) | *Lactuca sativa* | 93 % | 100 % | 4e-21 |
|  | [ABB52807.1](http://www.ncbi.nlm.nih.gov/protein/80971672?report=genbank&log$=prottop&blast_rank=117&RID=WPYDZ7VS01R) | *Helianthus annuus* | 93 % | 100 % | 7e-21 |
|  | [ADZ24709.1](http://www.ncbi.nlm.nih.gov/protein/325516262?report=genbank&log$=prottop&blast_rank=141&RID=WPXS5D2001R) | *Solanum pennellii* | 91 % | 100 % | 7e-20 |

**Supplementary Table 10.** Protein homology of VTE1 in olives involved in the biosynthetic pathway of vitamin E.

| **Gene** | **Accession Number** | **Plant Species** | **Percentage**  **Identity** | **Percentage**  **Similarity** | **E-value** |
| --- | --- | --- | --- | --- | --- |
| *VTE1* | [ABB52813.1](http://www.ncbi.nlm.nih.gov/protein/80971684?report=genbank&log$=prottop&blast_rank=1&RID=WMYDGFHS01R) | *Helianthus annuus* | 84 % | 100 % | 2e-19 |
|  | [ABB52812.1](http://www.ncbi.nlm.nih.gov/protein/80971682?report=genbank&log$=prottop&blast_rank=2&RID=WMYDGFHS01R) | *Helianthus annuus* | 84 % | 100 % | 2e-19 |
|  | [ADC91914.1](http://www.ncbi.nlm.nih.gov/protein/289186607?report=genbank&log$=prottop&blast_rank=7&RID=WMYDGFHS01R) | *Lactuca sativa* | 78 % | 100 % | 1e-18 |
|  | [KHF98490.1](http://www.ncbi.nlm.nih.gov/protein/728808751?report=genbank&log$=prottop&blast_rank=11&RID=WMYDGFHS01R) | *Gossypium arboreum* | 80 % | 100 % | 5e-18 |
|  | [KHG07989.1](http://www.ncbi.nlm.nih.gov/protein/728828546?report=genbank&log$=prottop&blast_rank=12&RID=WMYDGFHS01R) | *Gossypium arboreum* | 80 % | 100 % | 5e-18 |
|  | [AAP97931.1](http://www.ncbi.nlm.nih.gov/protein/33188419?report=genbank&log$=prottop&blast_rank=20&RID=WMYDGFHS01R) | *Eucalyptus gunnii* | 76 % | 100 % | 3e-17 |
|  | NP_001291340.1 | *Sesamum indicum* | 80 % | 100% | 1e-17 |
|  | [AIC85301.1](http://www.ncbi.nlm.nih.gov/protein/655438079?report=genbank&log$=prottop&blast_rank=34&RID=WMYDGFHS01R) | *Nicotiana tabacum* | 70 % | 100 % | 3e-16 |
|  | [BAH10644.1](http://www.ncbi.nlm.nih.gov/protein/219842174?report=genbank&log$=prottop&blast_rank=38&RID=WMYDGFHS01R) | *Hevea brasiliensis* | 72 % | 100 % | 7e-16 |
|  | [AHH29648.1](http://www.ncbi.nlm.nih.gov/protein/576317990?report=genbank&log$=prottop&blast_rank=45&RID=WMYDGFHS01R) | *Juglans regia* | 72 % | 100 % | 4e-15 |
|  | [KHN10209.1](http://www.ncbi.nlm.nih.gov/protein/734343086?report=genbank&log$=prottop&blast_rank=47&RID=WMYDGFHS01R) | *Glycine soja* | 72 % | 100 % | 5e-15 |
|  | [KHN18183.1](http://www.ncbi.nlm.nih.gov/protein/734366750?report=genbank&log$=prottop&blast_rank=48&RID=WMYDGFHS01R) | *Glycine soja* | 72 % | 100 % | 5e-15 |
|  | [XP_003636442.1](http://www.ncbi.nlm.nih.gov/protein/358344734?report=genbank&log$=prottop&blast_rank=53&RID=WMYDGFHS01R) | *Medicago truncatula* | 70 % | 100 % | 7e-15 |
|  | [KEH36047.1](http://www.ncbi.nlm.nih.gov/protein/657395879?report=genbank&log$=prottop&blast_rank=54&RID=WMYDGFHS01R) | *Medicago truncatula* | 70 % | 100 % | 8e-15 |
|  | [NP_567906.1](http://www.ncbi.nlm.nih.gov/protein/18418083?report=genbank&log$=prottop&blast_rank=67&RID=WMYDGFHS01R) | *Arabidopsis thaliana* | 69 % | 100 % | 1e-13 |
|  | [AFB74210.1](http://www.ncbi.nlm.nih.gov/protein/377657553?report=genbank&log$=prottop&blast_rank=70&RID=WMYDGFHS01R) | *Brassica napus* | 67 % | 100 % | 1e-13 |
|  | [AFB74209.1](http://www.ncbi.nlm.nih.gov/protein/377657551?report=genbank&log$=prottop&blast_rank=73&RID=WMYDGFHS01R) | *Brassica napus* | 67 % | 100 % | 2e-13 |
|  | [NP_001274927.1](http://www.ncbi.nlm.nih.gov/protein/568214337?report=genbank&log$=prottop&blast_rank=80&RID=WMYDGFHS01R) | *Solanum tuberosum* | 63 % | 100 % | 2e-12 |
|  | [ADZ24706.1](http://www.ncbi.nlm.nih.gov/protein/325516256?report=genbank&log$=prottop&blast_rank=81&RID=WMYDGFHS01R) | *Solanum pennellii* | 63 % | 100 % | 2e-12 |
|  | [KMZ57637.1](http://www.ncbi.nlm.nih.gov/protein/901794433?report=genbank&log$=prottop&blast_rank=84&RID=WMYDGFHS01R) | *Zostera marina* | 62 % | 100 % | 1e-11 |
|  | [ACD50891.1](http://www.ncbi.nlm.nih.gov/protein/188484539?report=genbank&log$=prottop&blast_rank=88&RID=WMYDGFHS01R) | *Phaseolus vulgaris* | 68 % | 82 % | 2e-09 |
|  | [ABE41800.1](http://www.ncbi.nlm.nih.gov/protein/91694297?report=genbank&log$=prottop&blast_rank=100&RID=WMYDGFHS01R) | *Triticum aestivum* | 58 % | 100 % | 4e-08 |

**Supplementary Table 11.** Protein homology of VTE4 in olives involved in the biosynthetic pathway of vitamin E.

| **Gene** | **Accession Number** | **Plant Species** | **Percentage**  **Identity** | **Percentage**  **Similarity** | **E-value** |
| --- | --- | --- | --- | --- | --- |
| *VTE4* | [AIA25703.1](http://www.ncbi.nlm.nih.gov/protein/639465136?report=genbank&log$=prottop&blast_rank=6&RID=WN010WB101R) | *Phyllostachys* *edulis* | 88 % | 97 % | 7e-29 |
|  | [NP_001275191.1](http://www.ncbi.nlm.nih.gov/protein/568215599?report=genbank&log$=prottop&blast_rank=11&RID=WN010WB101R) | *Solanum tuberosum* | 73 % | 100 % | 2e-17 |
|  | [EPS62543.1](http://www.ncbi.nlm.nih.gov/protein/527191910?report=genbank&log$=prottop&blast_rank=12&RID=WN010WB101R) | *Genlisea aurea* | 88 % | 75 % | 3e-17 |
|  | [AIA25704.1](http://www.ncbi.nlm.nih.gov/protein/639465138?report=genbank&log$=prottop&blast_rank=13&RID=WN010WB101R) | *Phyllostachys nidularia* | 90 % | 75 % | 3e-17 |
|  | [AIA25701.1](http://www.ncbi.nlm.nih.gov/protein/639465132?report=genbank&log$=prottop&blast_rank=14&RID=WN010WB101R) | *Fargesia nitida* | 90 % | 75 % | 4e-17 |
|  | [AAL36933.1](http://www.ncbi.nlm.nih.gov/protein/17224292?report=genbank&log$=prottop&blast_rank=18&RID=WN010WB101R) | *Perilla frutescens* | 88 % | 75 % | 8e-17 |
|  | [AEO80033.1](http://www.ncbi.nlm.nih.gov/protein/347326940?report=genbank&log$=prottop&blast_rank=19&RID=WN010WB101R) | *[Solanum lycopersicum](http://blast.ncbi.nlm.nih.gov/Blast.cgi#alnHdr_347326940)* | 71 % | 75 % | 8e-17 |
|  | [NP_001298117.1](http://www.ncbi.nlm.nih.gov/protein/908672126?report=genbank&log$=prottop&blast_rank=20&RID=WN010WB101R) | *[Solanum lycopersicum](http://blast.ncbi.nlm.nih.gov/Blast.cgi#alnHdr_347326940)* | 71 % | 75 % | 8e-17 |
|  | [NP_001233814.1](http://www.ncbi.nlm.nih.gov/protein/350538087?report=genbank&log$=prottop&blast_rank=21&RID=WN010WB101R) | *[Solanum lycopersicum](http://blast.ncbi.nlm.nih.gov/Blast.cgi#alnHdr_347326940)* | 71 % | 75 % | 8e-17 |
|  | [ADZ24710.1](http://www.ncbi.nlm.nih.gov/protein/325516264?report=genbank&log$=prottop&blast_rank=22&RID=WN010WB101R) | *Solanum pennellii* | 71 % | 75 % | 8e-17 |
|  | [ABE41795.1](http://www.ncbi.nlm.nih.gov/protein/91694287?report=genbank&log$=prottop&blast_rank=24&RID=WN010WB101R) | *Solanum tuberosum* | 71 % | 100 % | 9e-17 |
|  | [AIA25705.1](http://www.ncbi.nlm.nih.gov/protein/639465140?report=genbank&log$=prottop&blast_rank=27&RID=WN010WB101R) | *Phyllostachys nigra var. henonis* | 90 % | 75 % | 9e-17 |
|  | [CAI77219.2](http://www.ncbi.nlm.nih.gov/protein/156712232?report=genbank&log$=prottop&blast_rank=36&RID=WN010WB101R) | *Triticum aestivum* | 90 % | 75 % | 3e-16 |
|  | [AAZ67143.1](http://www.ncbi.nlm.nih.gov/protein/72256521?report=genbank&log$=prottop&blast_rank=37&RID=WN010WB101R) | *Triticum aestivum* | 90 % | 75 % | 3e-16 |
|  | [ABS76142.1](http://www.ncbi.nlm.nih.gov/protein/154354064?report=genbank&log$=prottop&blast_rank=53&RID=WN010WB101R) | *Elaeis oleifera* | 86 % | 75 % | 8e-16 |
|  | [AEU17779.1](http://www.ncbi.nlm.nih.gov/protein/358680766?report=genbank&log$=prottop&blast_rank=54&RID=WN010WB101R) | *Elaeis guineensis* | 86 % | 75 % | 9e-16 |
|  | [AAX63740.1](http://www.ncbi.nlm.nih.gov/protein/62115031?report=genbank&log$=prottop&blast_rank=59&RID=WN010WB101R) | *Medicago truncatula* | 86 % | 75 % | 2e-15 |
|  | [ABB52798.1](http://www.ncbi.nlm.nih.gov/protein/80971651?report=genbank&log$=prottop&blast_rank=66&RID=WN010WB101R) | *Helianthus annuus* | 85 % | 69 % | 5e-15 |
|  | [ABB52801.1](http://www.ncbi.nlm.nih.gov/protein/80971657?report=genbank&log$=prottop&blast_rank=67&RID=WN010WB101R) | *Helianthus annuus* | 85 % | 69 % | 5e-15 |
|  | [ABB52799.1](http://www.ncbi.nlm.nih.gov/protein/80971653?report=genbank&log$=prottop&blast_rank=68&RID=WN010WB101R) | *Helianthus annuus* | 85 % | 69 % | 5e-15 |
|  | [AGF92694.1](http://www.ncbi.nlm.nih.gov/protein/452076257?report=genbank&log$=prottop&blast_rank=69&RID=WN010WB101R) | *Zea mays subsp. mays* | 75 % | 85 % | 5e-15 |
|  | [ABB52800.1](http://www.ncbi.nlm.nih.gov/protein/80971655?report=genbank&log$=prottop&blast_rank=71&RID=WN010WB101R) | *Helianthus annuus* | 85 % | 69 % | 5e-15 |
|  | [ACS34775.1](http://www.ncbi.nlm.nih.gov/protein/239918837?report=genbank&log$=prottop&blast_rank=72&RID=WN010WB101R) | *Artemisia sphaerocephala* | 75 % | 85 % | 5e-15 |
|  | [ABE41798.1](http://www.ncbi.nlm.nih.gov/protein/91694293?report=genbank&log$=prottop&blast_rank=75&RID=WN010WB101R) | *Gossypium hirsutum* | 86 % | 75 % | 7e-15 |
|  | [KEH39886.1](http://www.ncbi.nlm.nih.gov/protein/657400790?report=genbank&log$=prottop&blast_rank=77&RID=WN010WB101R) | *Medicago truncatula* | 86 % | 75 % | 8e-15 |
|  | [ADC91915.1](http://www.ncbi.nlm.nih.gov/protein/289186609?report=genbank&log$=prottop&blast_rank=78&RID=WN010WB101R) | *Lactuca sativa* | 73 % | 85 % | 8e-15 |
|  | [AAY52459.1](http://www.ncbi.nlm.nih.gov/protein/66732623?report=genbank&log$=prottop&blast_rank=79&RID=WN010WB101R) | *Lotus japonicus* | 86 % | 75 % | 9e-15 |
|  | [AFJ49047.1](http://www.ncbi.nlm.nih.gov/protein/386926095?report=genbank&log$=prottop&blast_rank=81&RID=WN010WB101R) | *Carthamus tinctorius* | 83 % | 75 % | 9e-15 |
|  | [AGT16736.1](http://www.ncbi.nlm.nih.gov/protein/530278810?report=genbank&log$=prottop&blast_rank=82&RID=WN010WB101R) | *Saccharum hybrid cultivar R570* | 83 % | 75 % | 9e-15 |
|  | [AFO70130.1](http://www.ncbi.nlm.nih.gov/protein/397914236?report=genbank&log$=prottop&blast_rank=83&RID=WN010WB101R) | *Carthamus tinctorius* | 83 % | 75 % | 9e-15 |
