# Supplementary material for: Temporal analysis reveals a key role for VTE5 in vitamin E biosynthesis in olive fruit during on-tree development
Source: Front Plant Sci. 2015 Oct 21;6:871. doi: 10.3389/fpls.2015.00871 (PMC4617049; doi:10.3389/fpls.2015.00871)

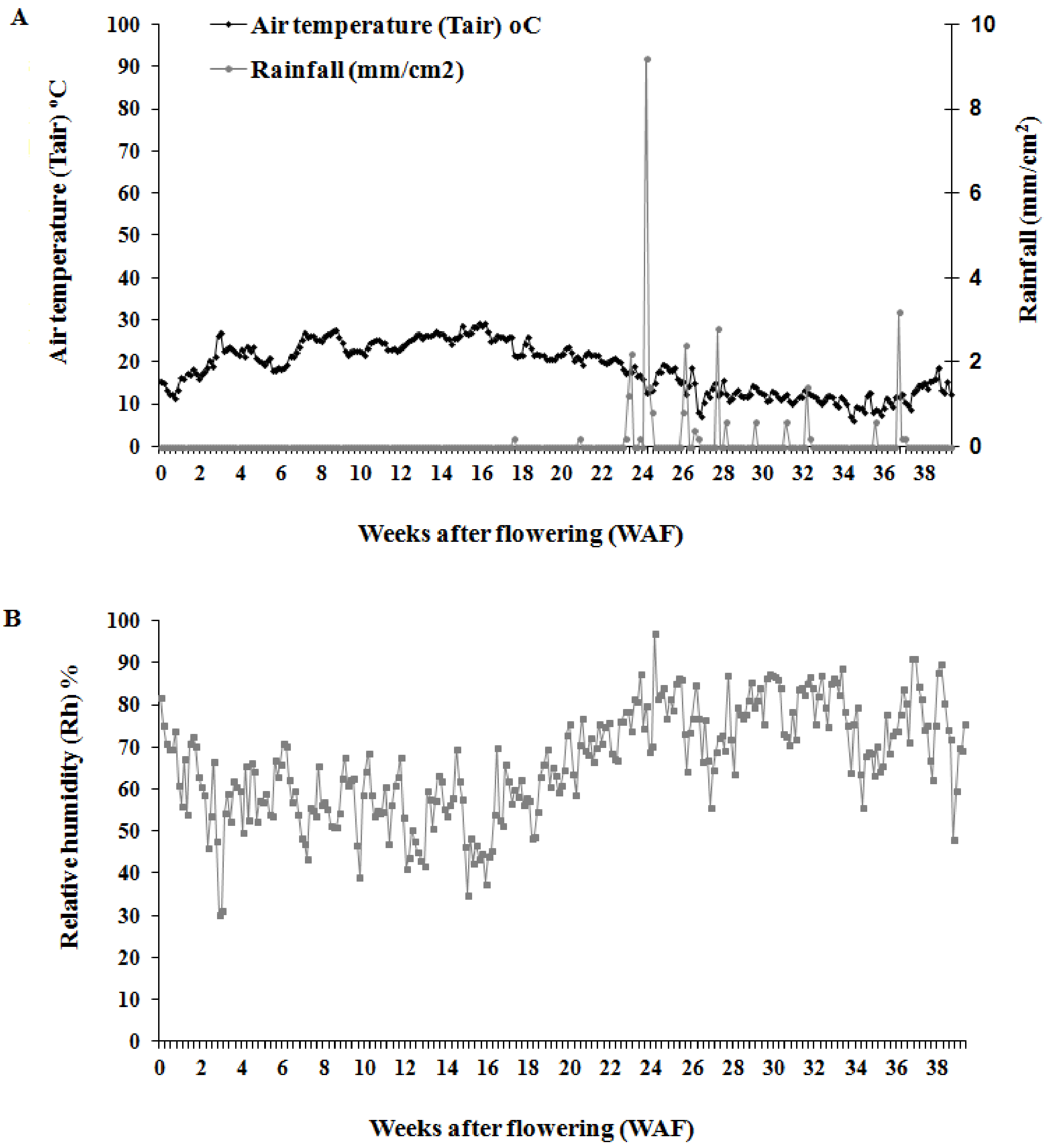

Supplementary Figure 1. A) Air temperature (Tair) °C and rainfall, B) Air relative humidity (RH) % during the 38 week period after flowering in the experimental orchard.

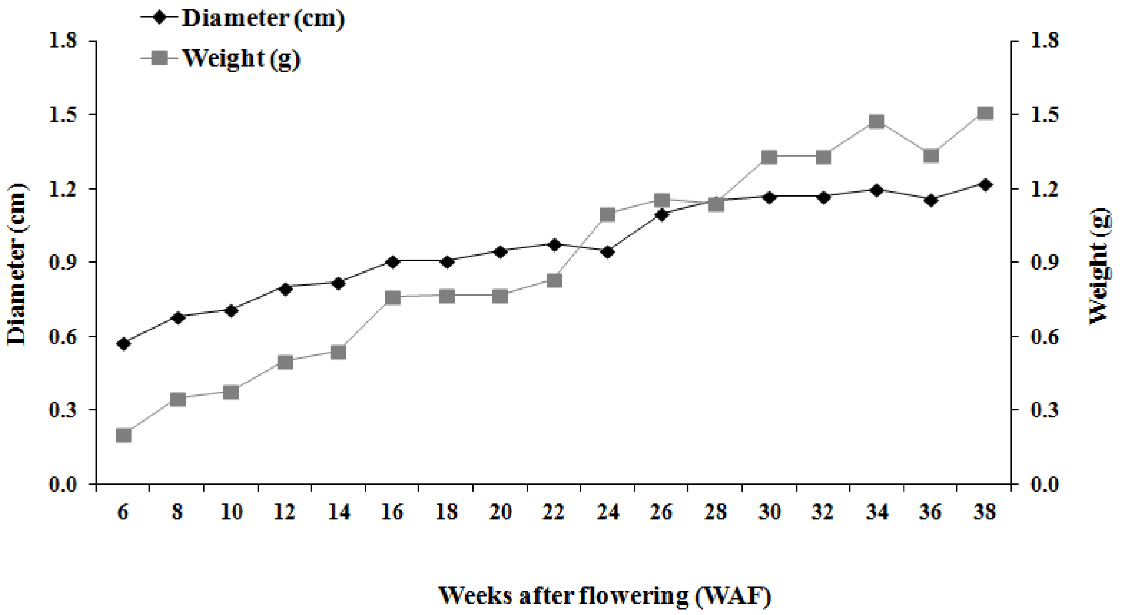

Supplementary Figure 2. Diameter (cm) and weight (g) of olive fruit during the 38 week period after flowering.

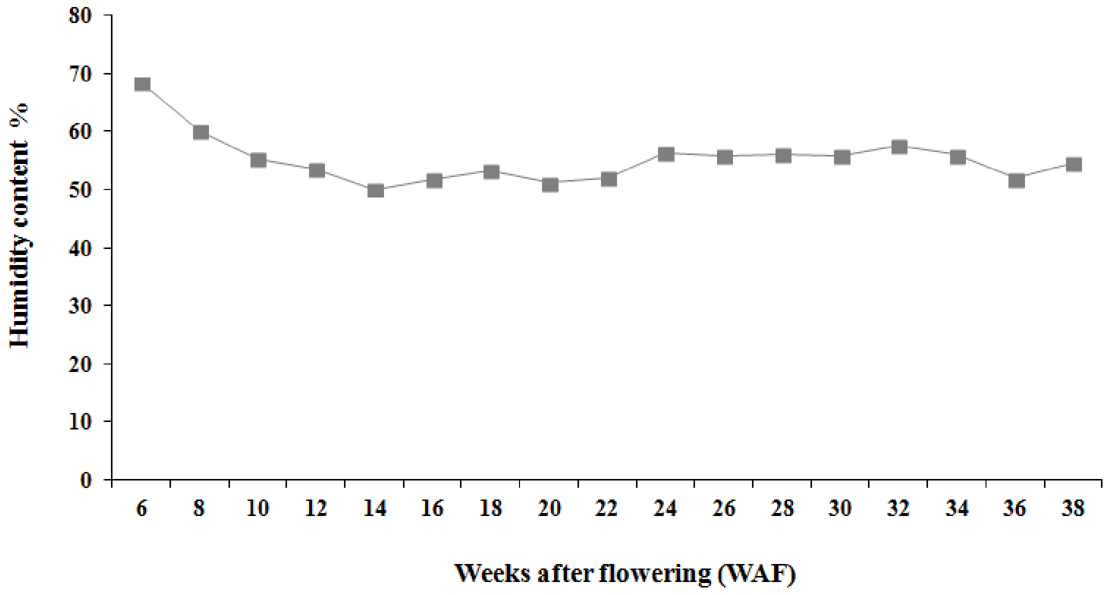

Supplementary Figure 3. Humidity content % during the 38 week period after flowering at the experimental orchard.

Supplementary Figure 4. Phylogenetic tree showed proteins that had similarity to the olive *VTE5* (and were also characterized as *VTE5*)

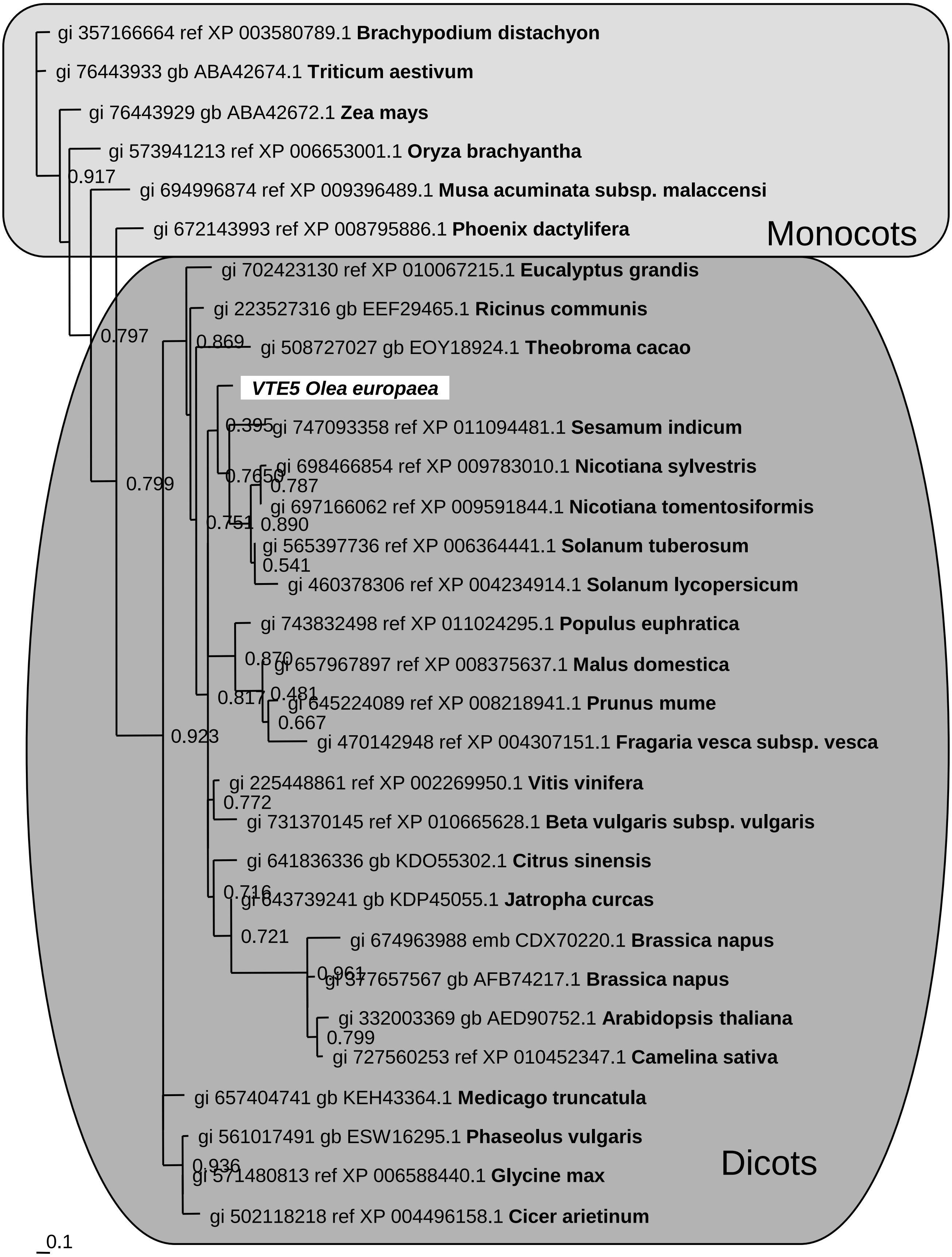

Supplementary Figure 5. Phylogenetic tree showed proteins that had similarity to the olive *HPPD* (and were also characterized as *HPPD*)

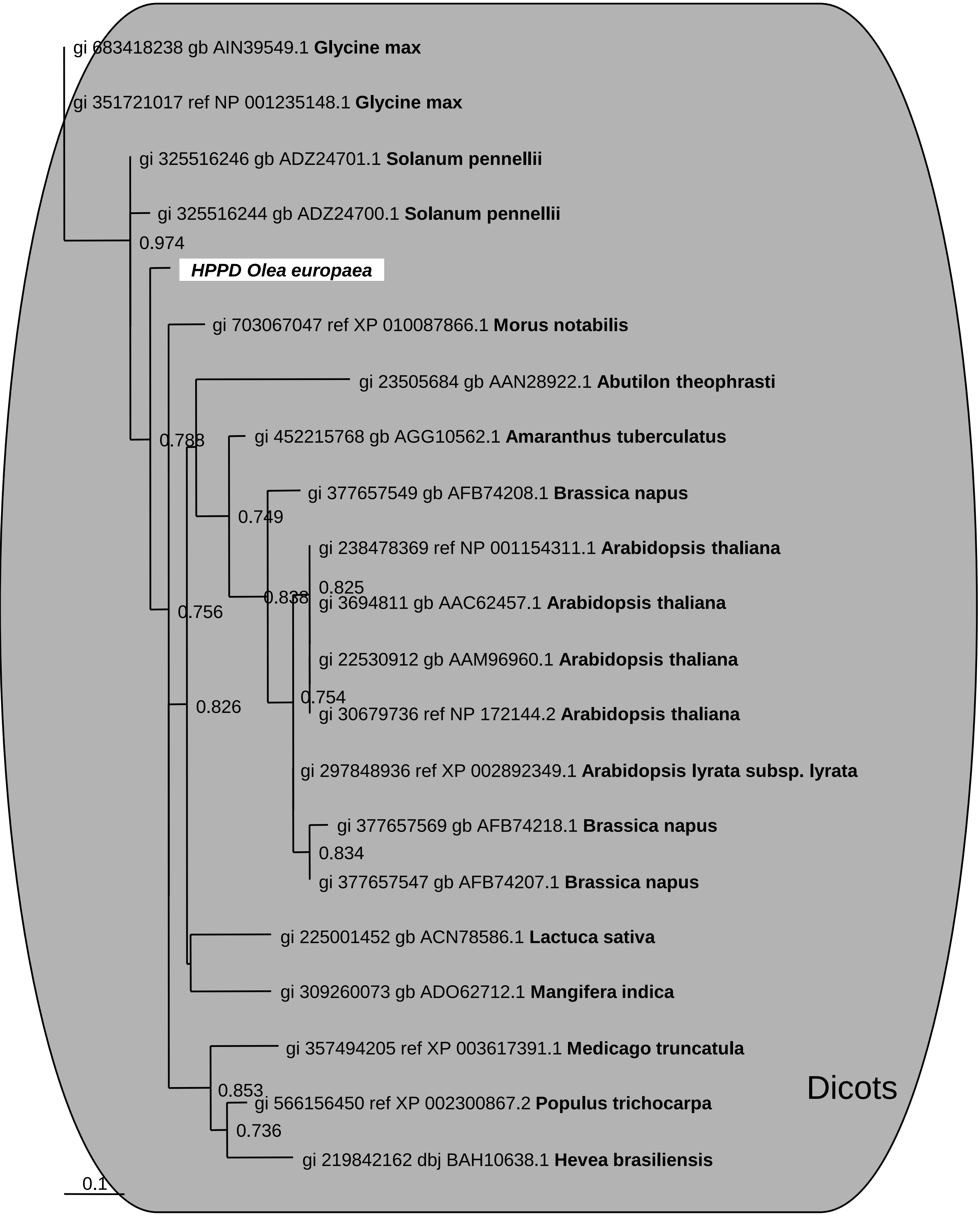

Supplementary Figure 6. Phylogenetic tree showed proteins that had similarity to the olive *VTE2* (and were also characterized as *VTE2*)

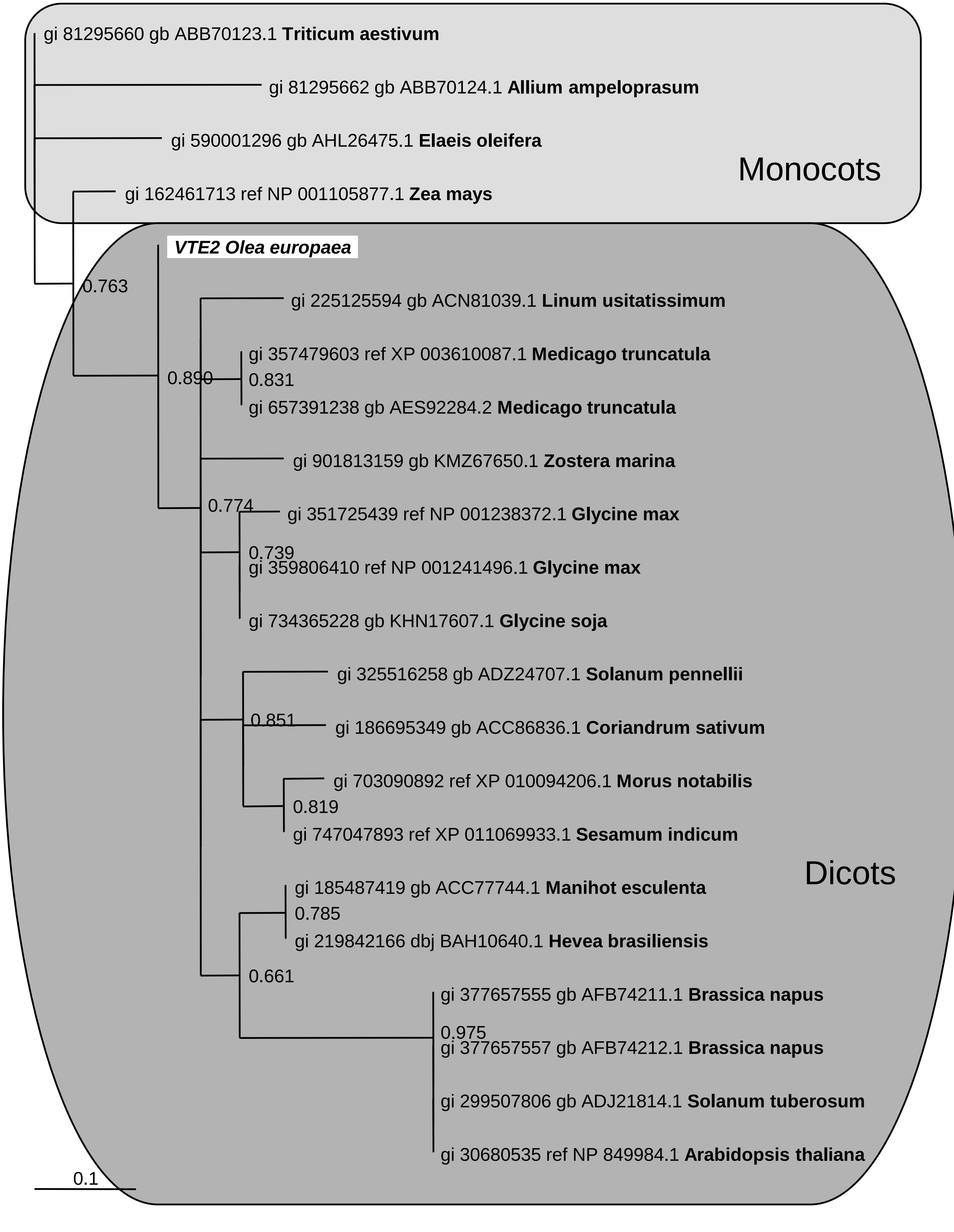

Supplementary Figure 7. Phylogenetic tree showed proteins that had similarity to the olive *HGGT* (and were also characterized as *HGGT*)

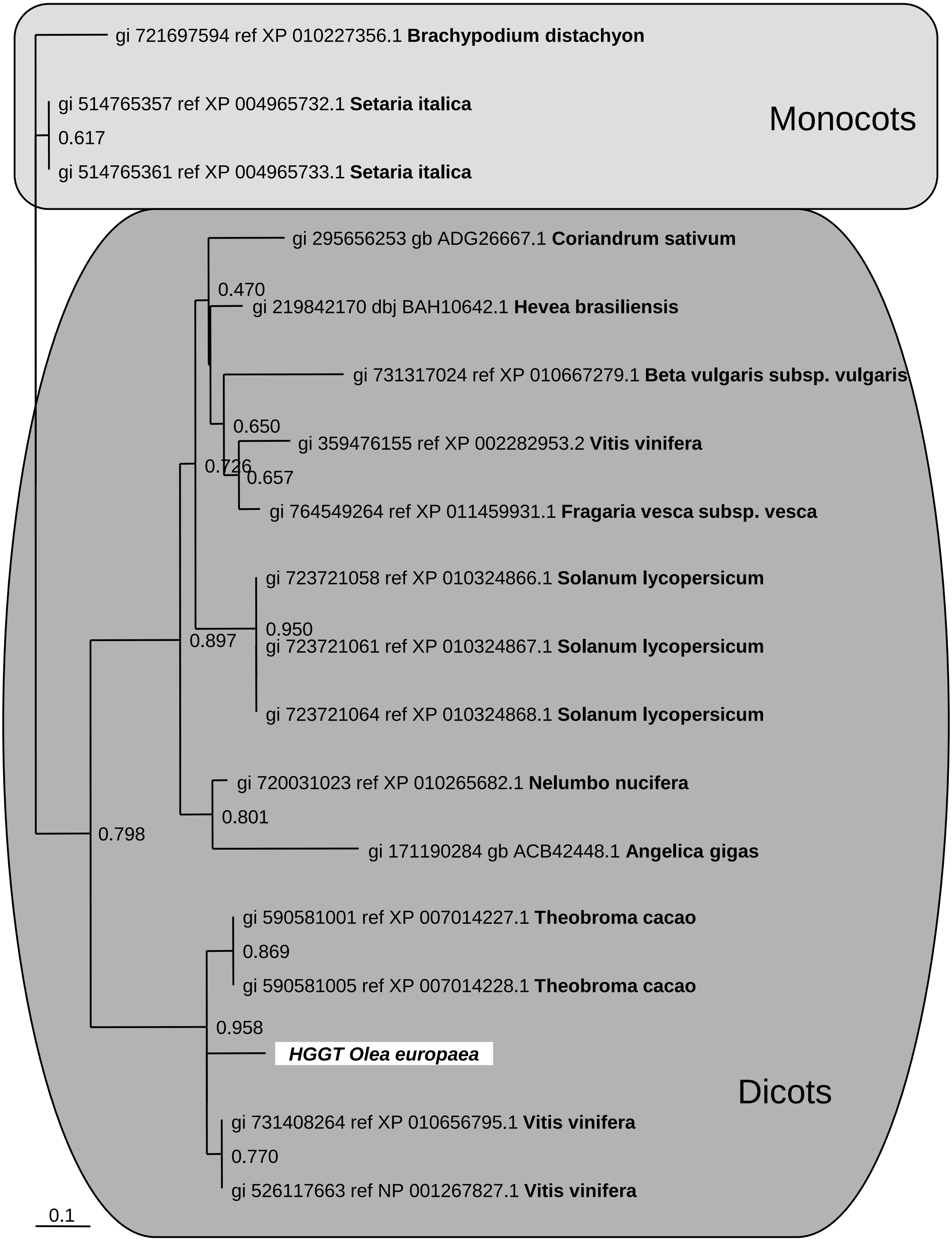

Supplementary Figure 8. Phylogenetic tree showed proteins that had similarity to the olive *VTE3* (and were also characterized as *VTE3*)

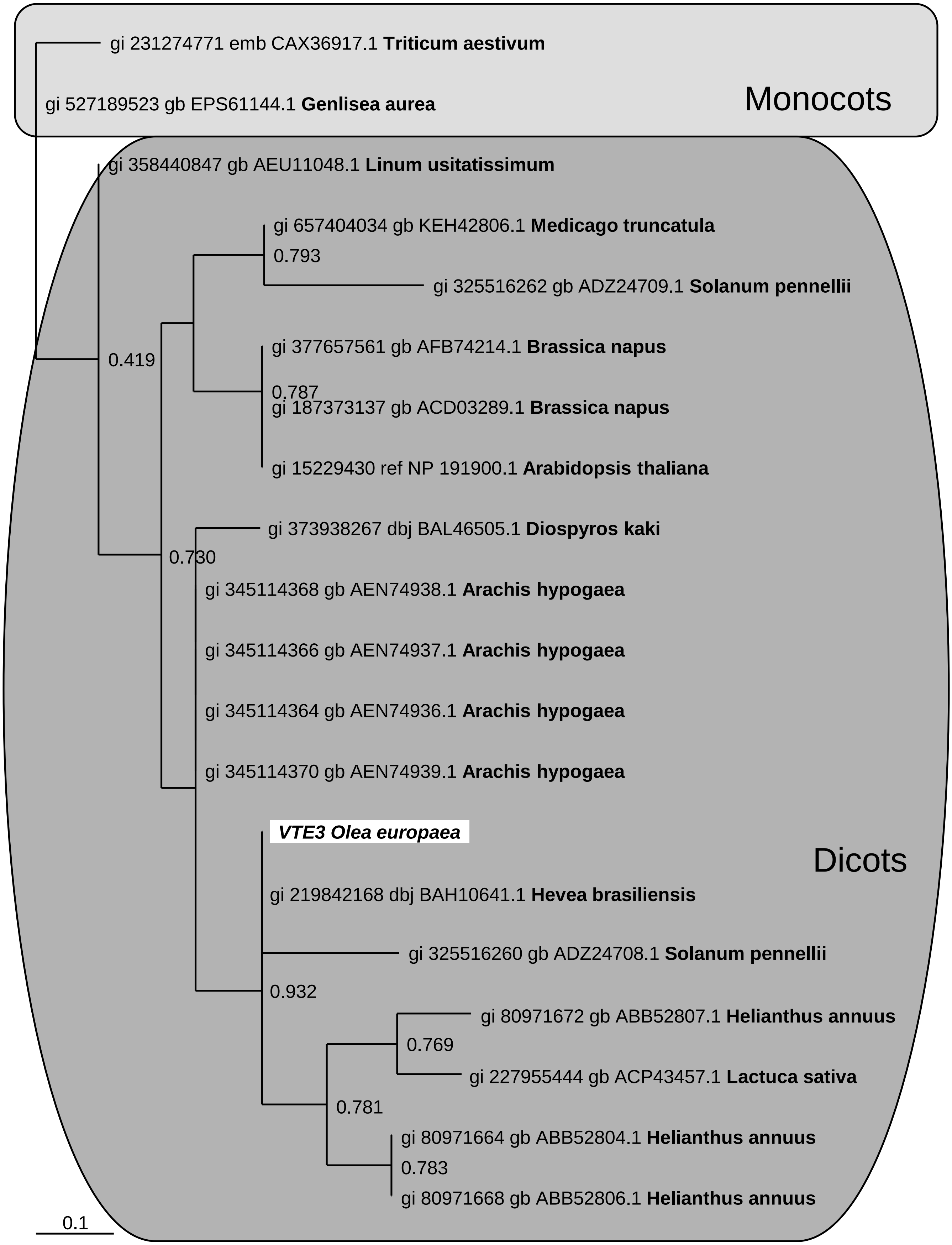

Supplementary Figure 9. Phylogenetic tree showed proteins that had similarity to the olive *VTE1* (and were also characterized as *VTE1*)

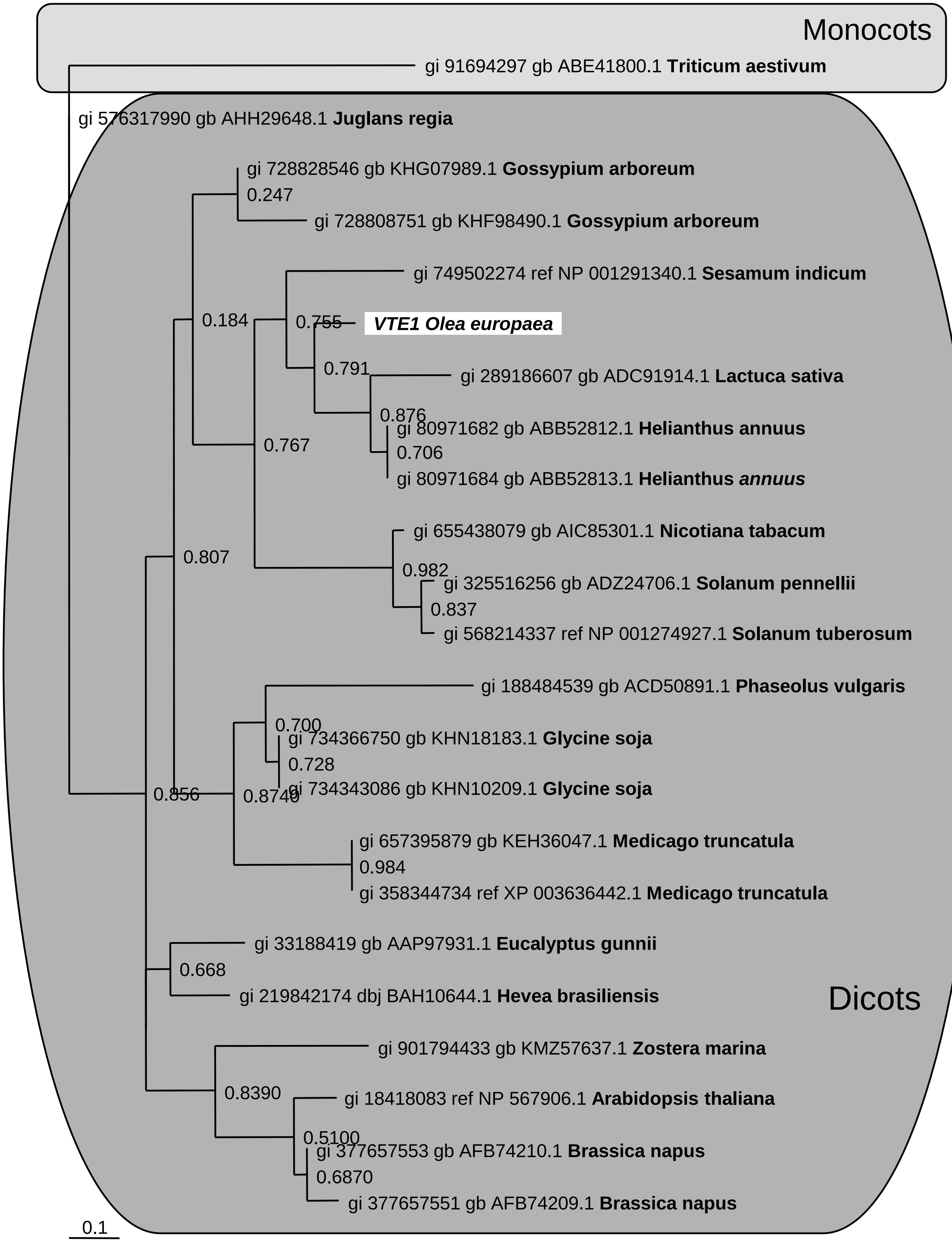

Supplementary Figure 10. Phylogenetic tree showed proteins that had similarity to the olive *VTE4* (and were also characterized as *VTE4*)

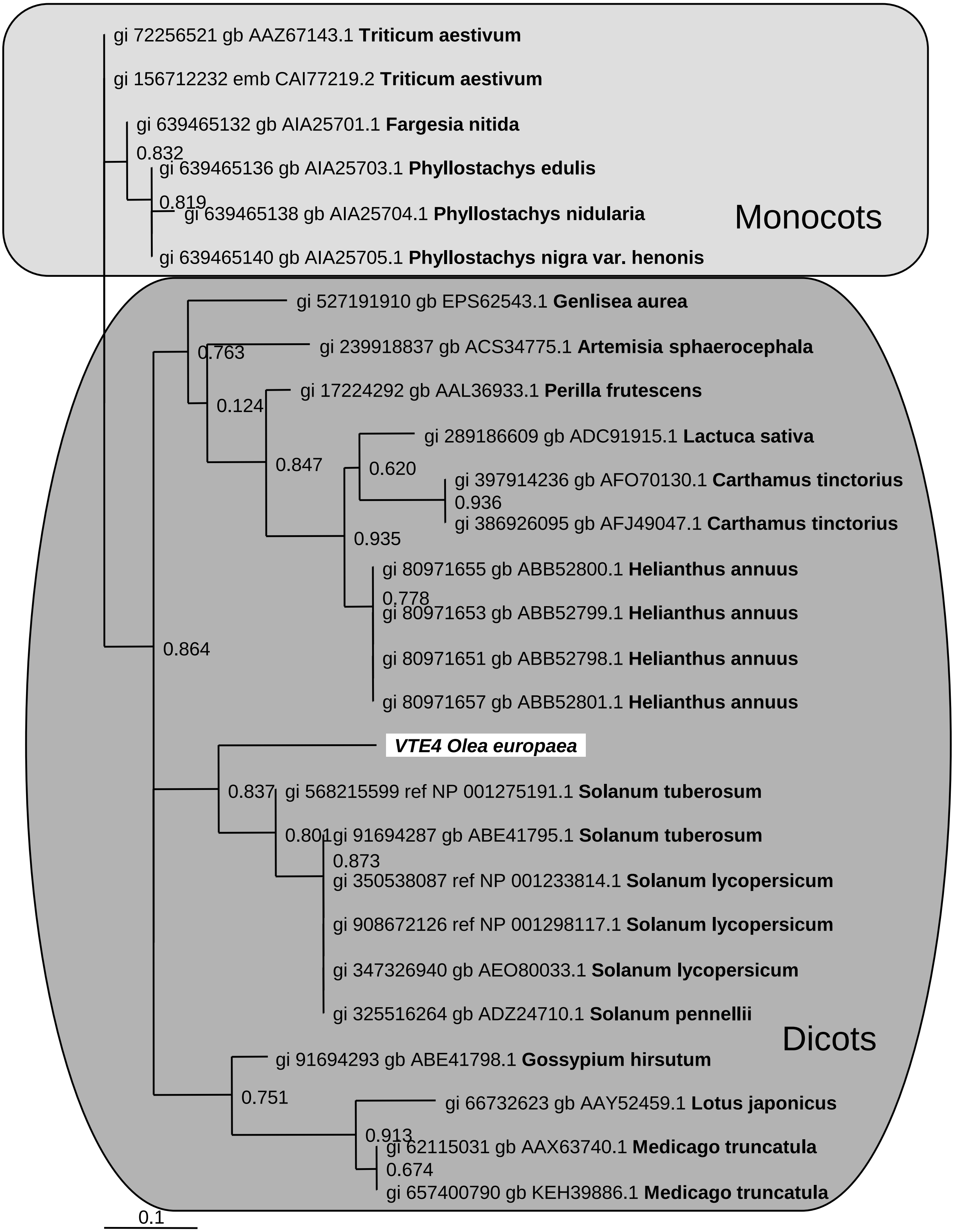

Supplement: Supplementary file 3 [file Presentation1.PDF]
